# Nanoparticle Platforms in Cancer Immunotherapy: A Critical Comparative Review of PLGA, Mesoporous Silica, Magnetic Nanoparticles, and Covalent Organic Frameworks

**DOI:** 10.3390/pharmaceutics18081019

**Published:** 2026-08-17

**Authors:** Sarfaraz K. Niazi

**Affiliations:** College of Pharmacy and Pharmaceutical Sciences, Washington State University, Spokane, WA 99202, USA; s.niazi@wsu.edu

**Keywords:** PLGA nanoparticles, mesoporous silica nanoparticles, magnetic nanoparticles, covalent organic frameworks, cancer immunotherapy, cGAS-STING, immune checkpoint blockade, clinical translation

## Abstract

**Background/Objectives**: Nanoparticle carriers can enhance cancer immunotherapy by improving tumor delivery, activating innate immune responses, and remodeling tumor microenvironments. This review evaluates four categories of formulations: poly(lactic-co-glycolic acid) (PLGA) nanoparticles, mesoporous silica nanoparticles (MSNs), magnetic or iron oxide nanoparticles (MNPs), and covalent organic frameworks (COFs). **Methods**: A reproducible PubMed audit identified 568 records. A rule-assisted title-and-abstract screen, followed by verification, removed 320 reviews, non-primary publications, and reports lacking qualifying in vivo formulation evidence. Of 248 potentially relevant reports, 15 primary studies were selected as representative examples; the remaining 233 were not classified as ineligible but were not selected as representative examples. These reports yielded 16 formulation-level records. One author conducted screening and extraction without protocol registration, duplicate review, or formal risk-of-bias scoring. **Results**: PLGA demonstrates the most robust polymer-level regulatory and manufacturing precedent; however, it remains limited by cargo instability, burst release, and challenges associated with process transfer. Biodegradable mesoporous silica nanoparticles (MSNs) facilitate pore-based protection and cytosolic delivery of cyclic dinucleotides, although their degradation and clearance are dependent on formulation specifics. Magnetic nanoparticles (MNPs) integrate magnetic targeting, imaging, and hyperthermia capabilities but necessitate formulation-specific magnetic characterization, field dosimetry, and repeated-dose safety assessments. Covalent organic frameworks (COFs) provide extensive stimulus-responsive and catalytic functionalities but exhibit the least mature evidence concerning biodegradation, scalable manufacturing, and independent reproducibility. Efficacy data across different studies were not pooled due to heterogeneity in models, schedules, comparators, and tumor-growth-inhibition formulas. **Conclusions**: The evidence does not endorse a universal platform ranking. Translation depends on standardized immune endpoints, explicit efficacy formulas, quantitative biodistribution assessments, mechanism-confirming experiments, repeat-dose toxicology studies, scalable manufacturing processes, and independent replication. The resulting evidence map serves as a descriptive and hypothesis-generating tool rather than a meta-analysis or clinical-priority scoring system.

## 1. Introduction

Cancer continues to be a leading cause of morbidity and mortality globally, with GLOBOCAN 2022 estimating approximately 20 million new cases and 9.7 million deaths worldwide [1]. Conventional therapeutic modalities, such as surgery, chemotherapy, and radiotherapy, form the cornerstone of oncological treatment; however, they are limited by systemic toxicity, tumor heterogeneity, acquired drug resistance, and an inability to generate sustained immune memory against residual disease [2].

Immune checkpoint blockade (ICB) targeting programmed death-1 (PD-1), its ligand (PD-L1), and cytotoxic T lymphocyte-associated antigen-4 (CTLA-4) has yielded sustained responses in select tumor types and biomarker-defined populations. However, a significant proportion of patients remain unresponsive, particularly those exhibiting poorly inflamed or immunosuppressive tumor microenvironments (TMEs) [3]. Adoptive cell therapies have expanded immunotherapy to include tumors resistant to checkpoint blockade alone [4], and personalized neoantigen vaccines have further diversified antigen-directed therapeutic strategies [5].

### 1.1. Approved Cancer Nanomedicines, Non-Oncology Nanoparticle Precedents, and Immunotherapeutic Nanoparticles

Nanoparticle therapeutics are already well-established in clinical oncology practice. Liposomal doxorubicin, which was introduced into clinical use in 1995, continues to serve as the benchmark for understanding how nanoscale formulations can modify biodistribution and toxicity profiles without affecting the pharmacological target [6]. Additionally, approved oncology nanoparticle products such as albumin-bound paclitaxel, liposomal irinotecan, the liposomal combination of cytarabine and daunorubicin, and liposomal vincristine have set significant regulatory precedents concerning chemistry, manufacturing, sterility, and characterization standards [7]. Currently, approximately 15 nanoparticle-based cancer nanomedicines have obtained regulatory approval worldwide [8].

A separate category must be kept distinct from that set. Ferumoxytol is an intravenous iron replacement product approved to treat iron deficiency anemia and is not an approved nanomedicine for cancer. It is discussed in this review solely as a regulatory and clinical safety precedent for an iron oxide nanoparticle formulation, and it is not included among approved oncology nanomedicines at any time. The same caution applies to nanoparticle imaging agents, which establish a precedent for the material and route but not for a therapeutic indication.

The distinction between these authorized products and the formulations analyzed herein is primarily mechanistic rather than semantic. It determines how their evidence should be interpreted. For the approved oncology nanomedicines mentioned above, the primary established function of the carrier is to modify payload disposition, exposure, and toxicity, whereas the therapeutic mechanism remains rooted in cytotoxicity against dividing cells. This should not be exaggerated. Some carriers also influence cellular uptake pathways, intracellular trafficking, or interactions with tumor-resident myeloid cells; these effects can contribute to the therapeutic activity. The key point is that such contributions are secondary to the primary change in disposition and are not the basis for the products’ approval.

Immunotherapeutic nanoparticles operate differently. The particle itself participates in pharmacology by directing cargo to antigen-presenting cells, delivering agonists to otherwise inaccessible intracellular sensors, converting cell death into an immunogenic event, or reprogramming stromal and myeloid populations. Three consequences follow. First, the relevant efficacy endpoints are immunological rather than purely cytoreductive, so tumor volume alone is an incomplete readout. Second, carrier immunogenicity, complement activation, and cytokine release shift from being tolerability nuisances to being mechanistically entangled with the intended effect. Third, regulatory precedent for a nanoparticle used as a delivery vehicle does not transfer to the same material used as an immune modulator, because the safety question changes from carrier tolerability to controlled immune activation. This distinction runs throughout the present review and explains why approved-product precedent is treated as relevant context rather than transferable evidence.

### 1.2. Current Clinical Trial Landscape for Nanoparticle Immunotherapy

Clinical trial activities within this domain should be categorized into three distinct levels, which are often mistakenly conflated. The initial level encompasses all nanomedical trials across various indications. A systematic classification of ClinicalTrials.gov revealed 4114 nanomedical trials out of over 500,000 registered studies, representing approximately 0.8% of all registered trials, with a 38% increase in registrations observed in recent years [9].

The second tier pertains to oncology nanomedicine. Oncology accounts for approximately 30% of nanomedical trials [9], and a registry survey confined to cancer nanomedicines identified 75 distinct products under clinical assessment across 190 trials, comprising 91 in phase 1, 78 in phase 2, and 21 in phase 3 [8]. These products mainly consist of cytotoxic delivery formulations rather than immunotherapeutic agents.

The third level, nanoparticle-based cancer immunotherapy specifically, is the level this review concerns, but neither cited registry analysis isolates it using a validated taxonomy. A broad registry query would mix nanoparticle-enabled immunotherapies with radiation, placebo, conventional checkpoint antibodies, and cytotoxic nanomedicines unless every record were adjudicated manually. An unsupported aggregate count is therefore not presented. Instead, the review reports the most current published registry statistics at the two reproducible levels above and separately identifies the clinical programs found for the four platform families: one PLGA immunomodulatory nanoparticle in a first-in-human phase I study, one magnetic hyperthermia system in early-feasibility evaluation, silica nanoparticles in first-in-human imaging studies but not immunotherapy trials, and no identified clinical COF program. These platform-specific observations are a lower bound rather than a registry census.

Attrition within this cohort is both severe and instructive. The success rate for completed Phase 2 trials with established outcomes was approximately 48%, whereas the success rate for Phase 3 trials decreased to approximately 14%, with failures primarily attributed to inadequate efficacy rather than toxicity [8]. Nanoparticle immunotherapy constructs represent a small and nascent segment within this already limited field. Among the four platform families examined in this review, the programs identified through the current literature search include one PLGA immunomodulatory nanoparticle in an initial-in-human Phase I study [10], one magnetic hyperthermia system in early feasibility evaluation under registration [11], and silica nanoparticles in initial-in-human imaging studies, although not in immunotherapy trials [12]. We identified no registered clinical program for covalent organic frameworks. These findings were obtained incidentally through the literature search rather than via a systematic registry inquiry; thus, the list reflects a minimum estimate and should not be considered comprehensive. Any assertion regarding translational readiness must thus be calibrated against clinical evidence of this magnitude rather than solely on preclinical activity.

### 1.3. Rationale for Platform Selection

Nanoparticles have emerged as effective delivery vectors capable of counteracting the immunosuppressive tumor microenvironment (TME), enhancing antigen presentation, and facilitating the co-delivery of multiple immunostimulatory agents. 

This review examines four nanoparticle platform families selected to encompass the design space rather than to survey it exhaustively: (1) PLGA nanoparticles, representing biodegradable synthetic polymers with an established record of safe excipient use; (2) MSNs, representing rigid inorganic porous carriers characterized by high loading capacity and tunable pore chemistry; (3) MNPs, representing theranostic materials with inherent imaging contrast and externally controllable physical actuation; and (4) COFs, representing crystalline porous organic frameworks with engineered molecular architecture and inherent photophysical properties. These four categories occupy distinct positions along the axes of degradability, rigidity, external controllability, and translational maturity, thereby providing a comprehensive basis for comparative evaluation.

Lipid nanoparticles, liposomes, exosomes, albumin nanoparticles, dendrimers, and metal–organic frameworks are outside this scope, and their exclusion carries no implication about clinical importance. The exclusions are deliberate and reasoned. Liposomes and albumin nanoparticles are excluded because their oncology evidence base is dominated by approved cytotoxic delivery products whose translational questions are largely settled and are qualitatively different from the questions facing immunotherapeutic carriers [6]. Lipid nanoparticles are excluded because the volume and maturity of the messenger RNA vaccine literature would dominate any comparative synthesis and would require a dedicated review to treat fairly [7]. Exosomes are excluded because they are biologically derived rather than synthetically defined, so composition control, characterization, and regulatory classification differ fundamentally from the four synthetic platforms compared here. Dendrimers are excluded because their in vivo evidence base for cancer immunotherapy remains too limited to support cross-platform comparison. Metal–organic frameworks are excluded from the primary comparison but are used as a deliberate contrast case for COFs in Section 2.4 because the two material classes share porosity and crystallinity but differ in the presence of coordinated metal centers, making the comparison mechanistically instructive [13,14].

**Figure 3:**
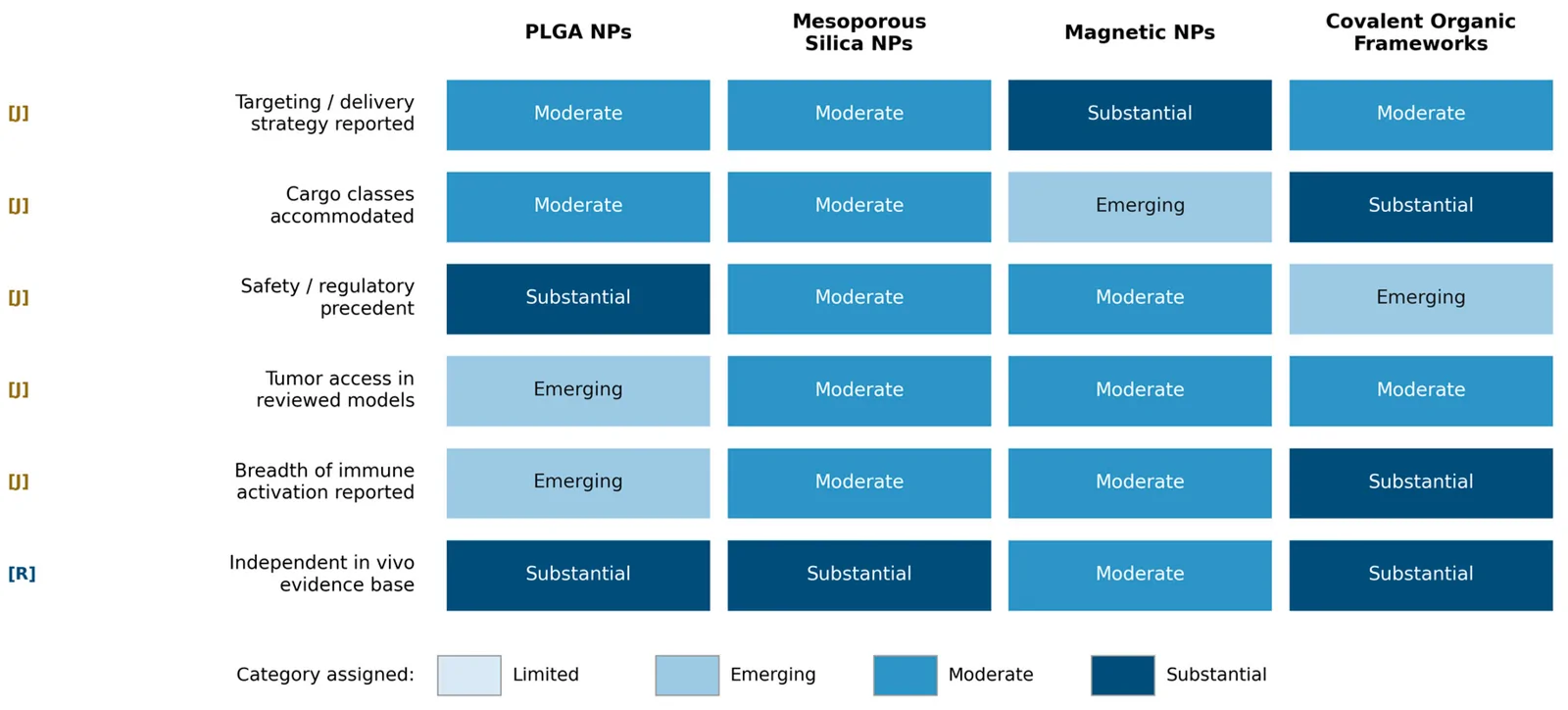
An author-assigned descriptive assessment of the reviewed evidence base for the four selected nanoparticle platform families. The rows are not ordered in a comparable manner and should not be interpreted as a single scale. The independent in vivo evidence base row, marked [R], is assigned according to the numerical counting rule outlined in Section Organization of the Evidence Map and Its Limitations, namely: Substantial for four or more independent reviewed in vivo formulations, Moderate for three, Emerging for two, and Limited for one or none. Readers can reproduce this classification by counting the qualifying rows in Table 2. The remaining five rows, marked [J], represent author-assigned qualitative judgments. These judgments are based on criteria that are not mutually comparable, including the physical distinctiveness of a targeting mechanism, the number of qualifying formulations, regulatory precedents outside the reviewed set, mechanistic breadth, and the depth of endpoint measurement. Different assessors applying the same descriptions to the same table could reasonably classify several cells differently. The ordered colour scale serves as a visual aid and is not an ordinal measurement. The cells are not aggregated into a composite score, do not constitute a meta-analysis, and do not rank platform efficacy. No tumor growth inhibition values are plotted because cross-study TGI values use different formulas, models, schedules, and comparators, making them unsuitable for direct comparison. All assignments are based on the 16 formulation-level records listed in Table 2, which were not systematically selected, and they do not imply greater precision than the evidence provides.

Despite the promise of these systems, significant obstacles remain, including limited tumor penetration, off-target toxicity, physicochemical instability, immunogenicity of carrier materials, and manufacturing scalability for clinical-grade production [15]. Due to variability in tumor models, routes of administration, payloads, dosages, combination regimens, and immune-endpoint depth across the available studies, cross-platform comparisons within this review generate hypotheses rather than definitive conclusions. A qualitative evidence map is provided in Table 1 and Table 2 and Figure 3 to organize findings and identify translational gaps.

### 1.4. Review Scope and Evidence-Selection Methods

This is a narrative critical review and representative evidence map, not a systematic review or meta-analysis. No protocol was registered, no independent duplicate screening or formal risk-of-bias assessment was conducted, and the selection of representative reports was based on judgment. The quantitative flow below is provided to make that selection transparent rather than to imply PRISMA compliance.

The quantitative evidence-selection audit was based exclusively on PubMed because it was the only database for which the complete search strategy and screening records were retained. PubMed was searched on 20 June 2026 for English-language records published from 1 January 2018 through 20 June 2026. The exact search expression was: (PLGA[tiab] OR “poly(lactic-co-glycolic acid)”[tiab] OR “mesoporous silica”[tiab] OR “iron oxide nanoparticle*”[tiab] OR “magnetic nanoparticle*”[tiab] OR “covalent organic framework*”[tiab] OR COF[tiab]) AND (“cancer immunotherapy”[tiab] OR “immune checkpoint”[tiab] OR cGAS-STING[tiab] OR “immunogenic cell death”[tiab] OR “tumor growth inhibition”[tiab]) AND English[lang] AND (“2018/01/01”[dp]: “2026/06/20”[dp]).

**Figure 1:**
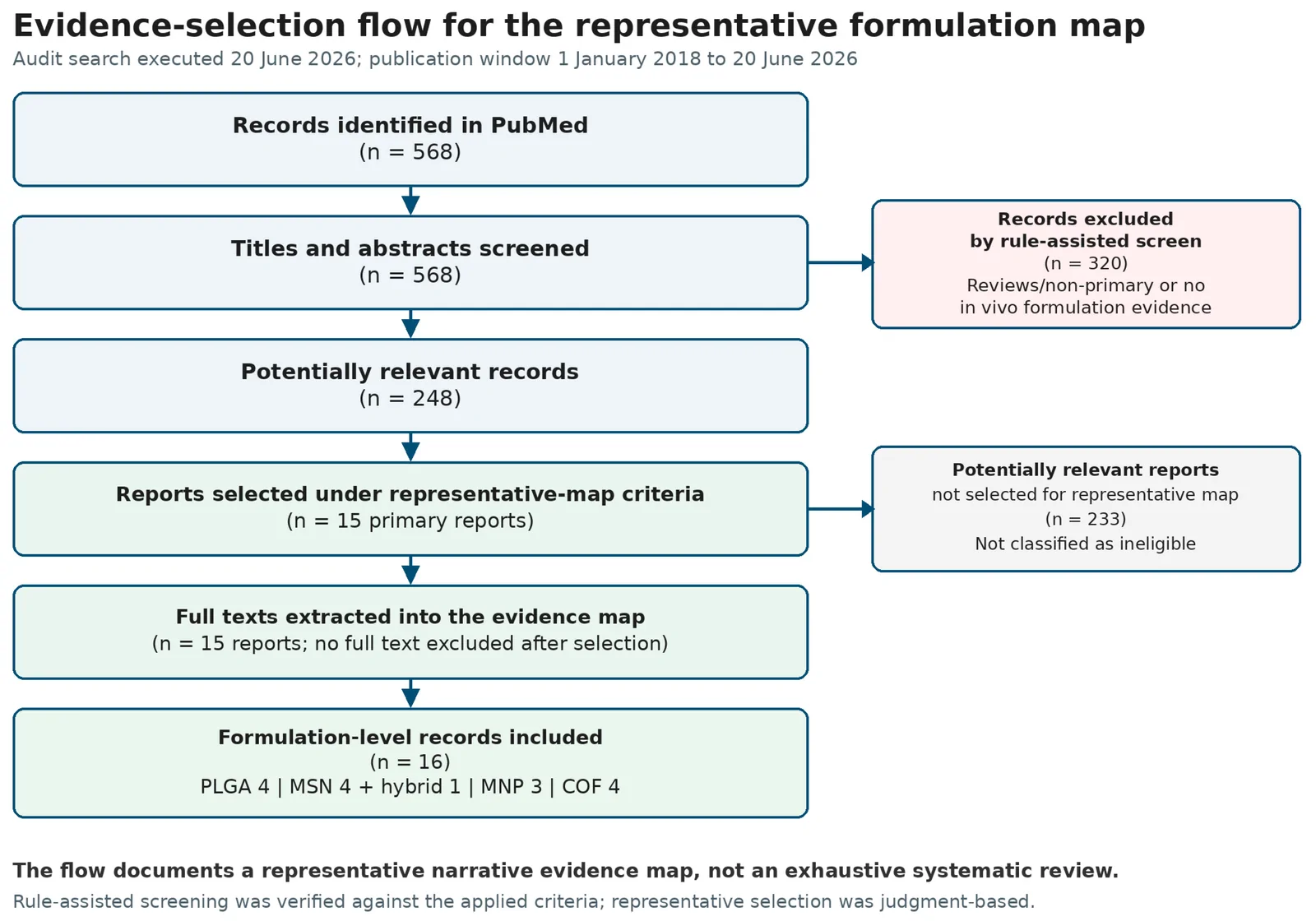
Evidence-selection flow for the representative formulation map. The audit search identified 568 PubMed records. A rule-assisted title-and-abstract screening, verified against the applied criteria, excluded 320 reviews, non-primary publications, and reports lacking in vivo formulation evidence. Of the 248 potentially relevant records, 15 primary reports were selected under the criteria for the representative map for full-text extraction; 233 potentially relevant reports were not classified as ineligible but were not selected as representative examples. The selected reports yielded 16 formulation-level records. The flow depicts a narrative evidence map and does not imply systematic review or PRISMA compliance.

**Figure 2:**
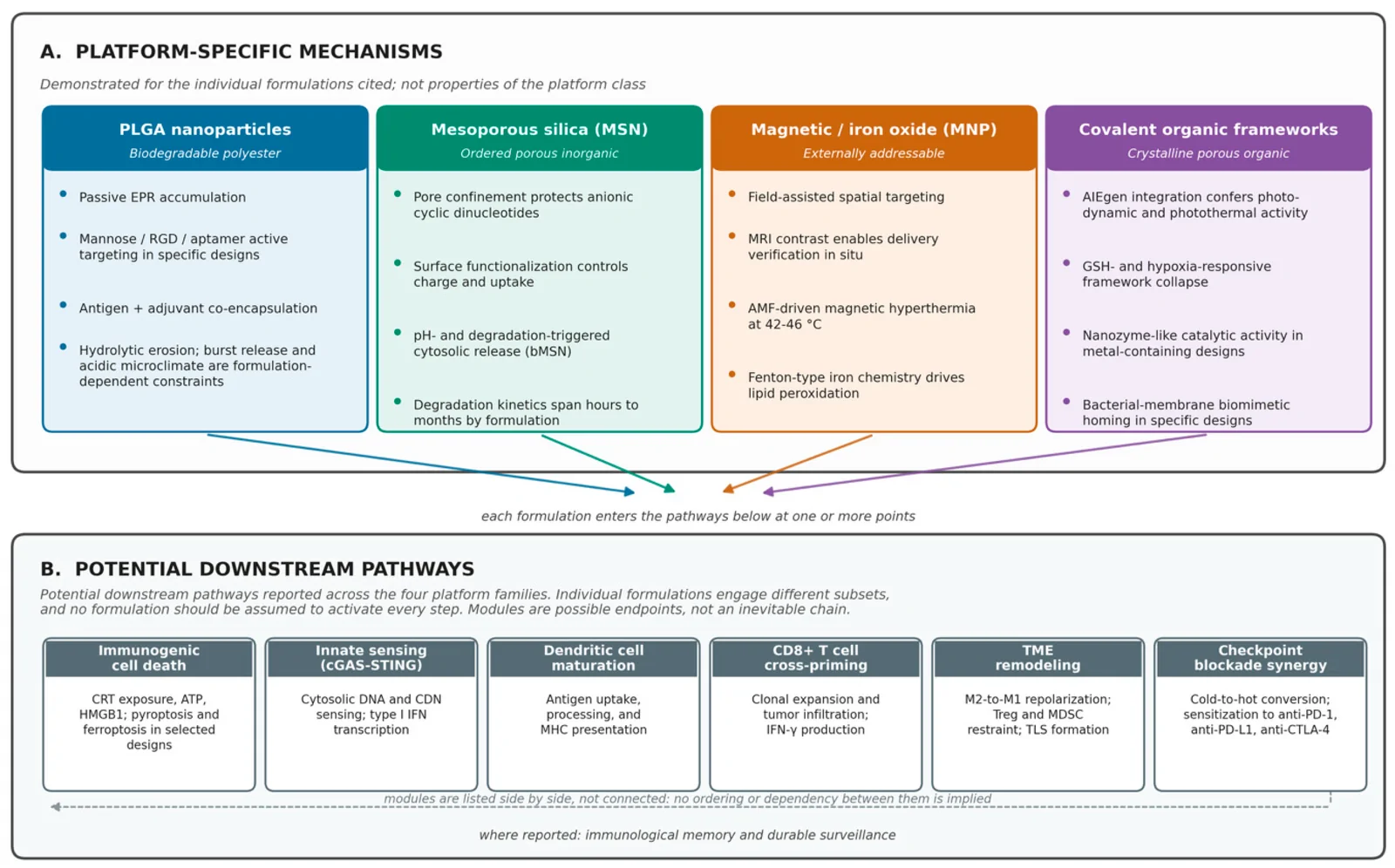
Representative Delivery Mechanisms and Immunological Cascades for the Four Selected Nanoparticle Platform Families in Cancer Immunotherapy. Panel (**A**) illustrates platform-specific mechanisms demonstrated for the individual formulations cited in Section 2.1, Section 2.2, Section 2.3 and Section 2.4, which should not be generalized to the entire platform class. PLGA nanoparticles depend on passive accumulation via the enhanced permeability and retention (EPR) effect, combined with receptor-ligand active targeting in specially engineered designs. Mesoporous silica nanoparticles (MSNs) protect cargo through pore-based encapsulation and surface functionalization and enable pH- or degradation-triggered release; biodegradable variants (bMSNs) enhance clearance. Magnetic nanoparticles (MNPs) enable field-assisted targeting, magnetic hyperthermia driven by alternating magnetic fields at temperatures between 42 and 46 degrees Celsius, and visibility in magnetic resonance imaging (MRI) for specific iron oxide formulations. Covalent organic frameworks (COFs) provide ligand- or stimulus-responsive release in certain designs, alongside photothermal, photodynamic, and nanozyme-like activities in AIEgen-integrated variants. Panel (**B**) presents potential downstream immunological modules reported across the four platform families. These modules are depicted side by side as possible endpoints, not as an obligatory sequence: individual formulations engage different subsets, with no implied order or dependency. Additionally, no module should be attributed to a formulation unless a directly measured endpoint is cited in a referenced study. Abbreviations include: AIEgen, aggregation-induced emission luminogen; AMF, alternating magnetic field; ATP, adenosine triphosphate; CDN, cyclic dinucleotide; cGAS, cyclic GMP-AMP synthase; CRT, calreticulin; CTLA-4, cytotoxic T lymphocyte-associated antigen-4; DC, dendritic cell; EPR, enhanced permeability and retention; GSH, glutathione; HMGB1, high-mobility group box 1; IFN, interferon; MDSC, myeloid-derived suppressor cell; MHC, major histocompatibility complex; MRI, magnetic resonance imaging; PD-1, programmed cell death protein 1; PD-L1, programmed death-ligand 1; RGD, arginine-glycine-aspartic acid; STING, stimulator of interferon genes; TLS, tertiary lymphoid structure; TME, tumor microenvironment; Treg, regulatory T cell.

The reproducible PubMed audit search identified 568 records. We screened titles and abstracts of all 568 records against the applied criteria. A rule-assisted first pass, followed by verification against publication type and the presence of in vivo formulation evidence, removed 320 reviews, non-primary publications, and reports without qualifying in vivo formulation evidence. Of the 248 potentially relevant records, 15 primary reports were selected for full-text extraction under the representative-map criteria described below; the remaining 233 were not classified as ineligible but were not selected as representative examples. No selected full text was subsequently excluded. The 15 reports yielded 16 formulation-level entries in Table 2. Screening, representative selection, and extraction were performed without a second reviewer, arbitration, or inter-rater statistics. Figure 1 summarizes the evidence-selection flow. A schematic overview of the representative delivery mechanisms and downstream immunological cascades is illustrated in Figure 2.

Here, we are eport the counts that can be verified from the manuscript itself. The formulation-specific evidence synthesis retained 16 formulation-level records drawn from 15 primary in vivo reports, comprising 4 PLGA formulations; 5 silica-based formulations, of which 4 are pure MSN constructs, and 1 is an MSN–iron oxide hybrid; 3 MNP formulations; and 4 COF formulations, all enumerated with their supporting citations in Table 2.

Section 8 and the abstract explicitly acknowledge single-reviewer screening as a limitation. Consequently, readers should interpret the evidence map with this context in mind.

The eligibility criteria outlined below were utilized during the screening process. These criteria were not registered beforehand, and no dated documentation confirms they were established before, rather than concurrent with, screening. Consequently, they are described as having been applied rather than being prespecified.

Reports were deemed eligible if they detailed a specific nanoparticle formulation within one of the four platform categories, assessed an immunotherapeutic mechanism or outcome in a preclinical or translational cancer model, and furnished adequate methodological details to attribute the findings to a particular formulation. Review articles served as background references and for cross-referencing but were not considered primary evidence for formulation-specific efficacy claims.

Studies lacking an immunotherapy endpoint, conference abstracts without complete data, and platforms outside the four selected families were excluded from the formulation-specific evidence synthesis and from Table 2. A single conference abstract reporting early clinical-stage magnetic hyperthermia observations is cited separately and explicitly labeled as clinical-context evidence from a conference abstract, rather than as a source of formulation-specific efficacy, consistent with this exclusion.

Due to the heterogeneity of the literature, representative rather than exhaustive studies were selected to exemplify the design space of each platform. The selection process was as follows: from the eligible pool, we preferred reports that included a defined comparator arm, at least one directly measured immune endpoint, and an in vivo antitumor outcome. Among reports meeting these criteria, priority was assigned to distinct mechanistic strategies over repeated instances of the same strategy, ensuring that each platform’s design space was represented rather than its most frequently published aspect. This procedure was conducted based on expert judgment rather than a scoring system, was not blinded, and was not documented on a case-by-case basis at the time of selection. Consequently, the resulting set of 15 reports is not reproducible solely based on the description provided and remains susceptible to selection bias. The descriptive evidence map does not account for these limitations.

Foundational, regulatory, and mechanistic references that establish background concepts, including reviews on immune checkpoints and adoptive cell therapy, STING species specificity, aptamer-targeting chemistry, and regulatory labels for approved products, were included as deliberate exceptions outside this time frame where required for context rather than for formulation-specific efficacy claims.

For each included study, the following fields were documented where available: platform and precise formulation, cargo, tumor model, route, dose, schedule, comparator arm, antitumor outcomes, including the TGI definition when provided, immune endpoints, and safety observations. Quantitative outcomes such as TGI are reported as obtained from the original sources. Because source studies use different TGI formulas (volume-based and weight-based), as well as different tumor models, schedules, and comparators, these values are not directly comparable across platforms and are therefore presented descriptively rather than pooled.

## 2. Nanoparticle Platforms: Mechanistic Overview

### 2.1. PLGA Nanoparticles

PLGA is a biodegradable polymeric excipient with extensive precedence in approved long-acting injectable drug products. Leuprolide acetate depot (Lupron Depot) is formulated as lyophilized microspheres containing a biodegradable lactic/glycolic acid copolymer [16], and risperidone long-acting injection (Risperdal Consta) utilizes a PLGA microsphere depot [17]. These products establish regulatory and manufacturing precedence for the polymer as a depot microsphere excipient. They do not establish precedent for PLGA nanoparticle immunotherapy formulations, each of which remains a distinct drug or biologic-device combination requiring product-specific CMC, sterility, residual-solvent, release, immunogenicity, biodistribution, and toxicology evaluation [2].

PLGA hydrolyzes to lactic acid and glycolic acid, which are endogenous or biocompatible metabolites cleared through normal metabolic and excretory pathways. The degradation rate depends on the lactide: glycolide ratio, molecular weight, crystallinity, end-group chemistry, particle size, and local pH [18].

#### 2.1.1. Protein Loading, Encapsulation Efficiency, and Burst Release

In the context of immunotherapeutic PLGA formulations, the primary concern lies not within the polymer itself but within the cargo. Protein and peptide antigens are subjected to four distinct stress environments during the double-emulsion fabrication process and subsequent release: the water–organic solvent interface during primary emulsification, which induces unfolding and aggregation; shear forces and cavitation during homogenization or sonication; dehydration throughout solvent removal and lyophilization; and the acidic microenvironment generated within the degrading matrix by the accumulation of lactic and glycolic acid oligomers, which can significantly lower the pH and promote deamidation, acylation, and aggregation of the encapsulated protein [19]. Consequently, the encapsulation efficiency for hydrophilic macromolecules is variable and highly dependent on the process. Reporting efficiency alone is insufficient as a quality attribute, as it measures only the mass entrapment rather than the immunologically intact antigen recovered. Therefore, vaccination formulations should also report antigen integrity and epitope preservation, in addition to loading, since high encapsulation efficiency coupled with a denatured antigen will lack immunological efficacy despite appearing satisfactory in analytical assessment.

The burst release compounds the problem. The release of PLGA generally proceeds through three distinct phases: an initial burst attributable to surface- and near-surface-associated cargo, a lag phase driven by diffusion through the intact matrix, and a subsequent phase driven by bulk erosion once the polymer’s molecular weight declines below the threshold for oligomer solubilization [18]. The initial burst is considered undesirable in depot vaccination, as it delivers a bolus of antigen and adjuvant that undermines the rationale for sustained exposure typical of depot formulations. Additionally, for potent innate agonists, this can induce a systemic cytokine spike. The magnitude of the burst is influenced by factors such as particle size and porosity, surface cargo adsorption, drug loading, and processing conditions, rather than by the polymer’s identity. Consequently, this parameter must be regulated on a formulation-by-formulation basis, as it cannot be inferred solely from the polymer specifications.

#### 2.1.2. Manufacturing Scale-Up

No PLGA nanoparticle cancer-immunotherapy product has been approved in any jurisdiction, nor has any PLGA nanoparticle formulation reached the global market for any indication, in contrast to the approved PLGA microsphere depot products outlined above [20]. The distinction lies between nanoparticles and microsphere depots, not between polymers. The primary obstacle for the nanoparticle format is related to manufacturing challenges rather than biological considerations. Laboratory-scale preparation methods, such as batch sonication, nanoprecipitation, or solvent evaporation, do not directly transfer to industrial-scale production, as the energy input, mixing regime, and solvent removal kinetics that influence particle size distribution and encapsulation efficiency all vary with vessel geometry. Additionally, downstream processes pose further difficulties: purification must eliminate residual surfactant and solvent without destabilizing the colloid; terminal sterilization is generally incompatible with polymeric nanoparticles, necessitating validation of sterile filtration or aseptic processing; and lyophilization requires cryoprotectant optimization to prevent irreversible aggregation [20]. Continuous manufacturing approaches utilizing inline sonication have successfully reproduced the colloidal and functional properties of laboratory-scale immunotherapeutic PLGA nanovaccines at an industrial scale. This shows the challenge is manageable; however, process transfer requires dedicated development rather than simple proportional scaling [21]. Consequently, any clinical development strategy for a PLGA immunotherapy nanoparticle should regard process definition as a critical-path activity, rather than as a downstream manufacturing task.

#### 2.1.3. Targeting, Cargo, and Representative Preclinical Evidence

PLGA nanoparticle formulations utilize passive accumulation through the enhanced permeability and retention (EPR) effect, complemented by receptor-ligand-mediated active targeting in specific configurations. Mannosylation of the PLGA surface enhances mannose receptor-mediated uptake by antigen-presenting cells, as demonstrated directly in mannosylated PLGA cancer vaccines [22]. Modification with arginine-glycine-aspartic acid (RGD) peptides, enabling alpha-v beta-3 integrin-targeted delivery, is exemplified in an RGD-functionalized lipid/PLGA nanocomplex [23]. Conjugation to the AS1411 aptamer confers nucleolin-directed recognition, as evidenced in AS1411-tagged PLGA-lecithin-PEG nanoparticles [24]. The ligand chemistry and targeting behavior should be ascribed to the specific formulation in which each was demonstrated, rather than being generalized to the PLGA class.

The spectrum of cargoes incorporated into PLGA formulations is extensive, encompassing metabolic inhibitors such as the indoleamine 2, 3-dioxygenase 1 (IDO1) inhibitor 1-methyl-tryptophan (1-MT), Toll-like receptor (TLR) agonists including R848 (which activates TLR7/8) and poly I:C (which activates TLR3), immune checkpoint antibodies such as anti-PD-1 and anti-PD-L1, tumor cell lysate antigens, and photosensitizers such as IR780 [25,26]. In studies utilizing mannosylated PLGA vaccines that co-deliver tumor cell lysate and poly I:C, a two- to three-fold enhancement in survival was observed within syngeneic breast cancer models, accompanied by elevated levels of interferon-gamma (IFN-gamma), a reduction in lung metastases, and increased infiltration of immune cells into treated tumors [22]. In a sonodynamic approach, PLGA nanoparticles coated with M1 macrophage membranes and co-loaded with IR780 and catalase, in combination with anti-PD-L1, impeded 4T1 metastasis in a preclinical model [25]. These findings highlight specific formulation strategies and should not be generalized to PLGA nanoparticles.

#### 2.1.4. Clinical Status of PLGA-Based Cancer Vaccines

The PLGA-based immunomodulatory nanoparticle PRECIOUS-01, which co-encapsulates the tumor antigen NY-ESO-1 and the invariant natural killer T (iNKT) cell activator IMM60 (threitolceramide-6, a synthetic alpha-galactosylceramide analog), has advanced to a first-in-human phase I dose-escalation clinical trial involving patients with NY-ESO-1-positive advanced cancers (ClinicalTrials.gov NCT04751786) [10]. This represents the sole such initiative identified in the present review, which was conducted as a literature search rather than through a systematic registry inquiry; consequently, the assertion reflects non-identification rather than proven exclusivity. A registry search specifying a date and search criteria would be necessary to confirm the absence of other programs. Broader claims that multiple PLGA-based cancer vaccine programs are currently in phase I or phase II would require specific trial references and are not asserted herein. It is important to recognize the asymmetry this introduces although PLGA possesses the most extensive excipient history and the broadest range of preclinical cargo among the four platforms, its clinical immunotherapy evidence is based on a single registered program [27].

### 2.2. Mesoporous Silica Nanoparticles (MSNs)

MSNs exhibit organized mesoporous structures of the MCM-41 and SBA-15 types, featuring adjustable pore diameters ranging from 2 to 50 nm and substantial surface areas between 500 and 1000 m^2^/g. This facilitates a high loading capacity for immunostimulatory agents [28]. Biodegradable variants (bMSNs) are designed with framework-labile silica bonds that degrade under physiological conditions.

#### 2.2.1. Biodegradation Kinetics, Tissue Persistence, and Immunotoxicity

Silica degradation occurs through the hydrolysis of siloxane bonds, resulting in soluble silicic acid that is subsequently excreted via the renal system. The reaction kinetics are not intrinsic to silica as a material; rather, they depend significantly on the specific particle characteristics. These kinetics can vary across several orders of magnitude depending on factors such as condensation degree, framework doping, particle size, pore architecture, surface area, and surface functionalization [29]. Highly condensed, nonporous silica may persist for months, whereas organosilica and framework-doped constructs designed with hydrolytically or redox-cleavable bridges may dissolve within hours to days [29]. Consequently, formulations intended for repeated dosing must establish individual degradation half-lives and silicic acid excretion balances, as the label “biodegradable MSN” does not convey any quantitative information at the platform level.

Long-term accumulation represents a relevant safety concern. Particles that do not undergo complete dissolution are predominantly cleared by hepatic and splenic mononuclear phagocytes, where their persistence can support granulomatous and fibrotic responses upon repeated exposure. Immunotoxicity associated with amorphous silica is distinct from that of crystalline silica and should not be conflated: the relevant endpoints for amorphous silica include complement activation, hypersensitivity-type infusion reactions, hemolysis caused by silanol interaction with erythrocyte membranes, inflammasome activation following phagolysosomal uptake, and inflammatory responses that depend on dose, particle size, morphology, and surface chemistry [30]. Surface silanol density serves as the primary determinant of hemolytic and membranolytic activity, indicating that surface passivation is a critical safety parameter rather than merely a formulation convenience [30]. For an immunotherapeutic mesoporous silica nanoparticle (MSN), the complexity is heightened because the innate pathways responsible for toxicity also contribute to the desired pharmacological effects. Consequently, the therapeutic window must be defined by measured immune endpoints rather than tolerability alone.

Silica has nonetheless reached the clinic. Ultrasmall (below 10 nm) cRGDY-functionalized silica particles labeled with iodine-124 completed a first-in-human study in metastatic melanoma under an investigational new drug application, demonstrating favorable safety and renal clearance [12]. That precedent is instructive but narrow: it applies to ultrasmall renally cleared particles used as imaging agents, not to mesoporous carriers in the 50 to 200 nm range delivering immune agonists on a repeat-dose schedule.

#### 2.2.2. STING Agonist Delivery and Representative Evidence

Biodegradable and cationically modified MSN formulations are highly suitable for the cytosolic delivery of anionic STING agonists such as cyclic dinucleotides (CDNs), as pore confinement safeguards CDNs from phosphodiesterase-mediated degradation. Additionally, pH-triggered dissolution of bMSN facilitates the release of cargo within the cytosol near cyclic GMP-AMP synthase (cGAS). The STING agonist cargoes encompass CDNs including c-di-GMP, c-di-AMP, cyclic GMP-AMP (cGAMP), and cyclic di-AMP analogs (CDAs), along with synthetic CDN analogs. Although the non-CDN agonist DMXAA has been employed in murine models, it is important to note that mouse STING, unlike human STING, binds and signals in response to DMXAA, thereby limiting its translational applicability. The performance of MSNs is influenced by factors such as pore architecture, surface charge, degradation kinetics, and endosomal escape efficiency, rather than being an inherent characteristic of the platform.

In a representative study, bMSN-delivered STING agonists demonstratyesed antitumor efficacy accompanied by prolonged survival in murine melanoma models [31]. CDA-Mn@bMSNs, which co-encapsulate CDNs and manganese to enhance STING signaling via cGAS sensitization and the release of endogenous mitochondrial DNA (mtDNA), exhibited significant therapeutic results against large, established orthotopic melanoma, characterized by systemic immune activation and improved checkpoint blockade efficacy [32]. Mitochondria-targeted manganese-based MSN platforms combined with anti-PD-L1 therapy effectively suppressed tumor growth in poorly immunogenic 4T1 triple-negative breast cancer (TNBC) models [33]. These findings are specific to the formulations investigated. It is important to note that reported tumor penetration varies according to formulation, influenced by factors such as particle size, surface chemistry, administration route, tumor model, and measurement technique, rather than representing an inherent property of MSNs.

The clinical counterweight should be stated directly and plainly. The first-generation free CDN STING agonist ADU-S100 (MIW815), administered intratumorally in 47 patients, was well tolerated and produced measurable systemic immune activation but yielded a single partial response [34], and combination with the PD-1 inhibitor spartalizumab in 106 patients produced minimal efficacy in refractory disease and was not advanced to phase II or III [35]. Whether nanoparticle-enabled delivery overcomes the pharmacological limitations identified in those trials, principally rapid clearance from the injection site, restricted cytosolic access, and inadequate distribution within the tumor, is the specific hypothesis that MSN-STING programs must test. It has not yet been tested clinically.

### 2.3. Magnetic Nanoparticles (MNPs)

Magnetic nanoparticle (MNP) systems primarily comprise iron oxide cores, such as Fe_3_O4 and gamma-Fe_2_O_3_, which may demonstrate superparamagnetic properties. These properties facilitate their use as contrast agents in magnetic resonance imaging (MRI) and enable hyperthermia treatment driven by alternating magnetic fields (AMFs) [36]. Superparamagnetism is dependent on particle size rather than chemical composition; magnetite and maghemite particles exhibit superparamagnetic behavior at ambient temperature only when their diameter is below a critical threshold of approximately 20 to 30 nanometers. Particles exceeding this size threshold tend to stabilize as single-domain ferrimagnets with remanent magnetization, and those above approximately 80 nanometers tend to display multidomain properties [37]. Consequently, the designation “superparamagnetic iron oxide nanoparticle” (SPION) applies exclusively to particles with core sizes below this critical limit. In contrast, coated or hybrid structures with larger cores or those possessing hydrodynamic diameters significantly exceeding their core dimensions should be described simply as iron oxide nanoparticles, unless magnetization measurements explicitly confirm superparamagnetic behavior.

Ferumoxytol (Feraheme) has received approval from the U.S. Food and Drug Administration as an iron replacement therapy for iron deficiency anemia in adults. This indication was expanded in 2018 beyond its original use in the chronic kidney disease population to include adults who are intolerant of or unresponsive to oral iron supplementation. Additionally, it has been explored preclinically as an imaging agent and as an immunomodulatory agent capable of directing tumor-associated macrophages towards a pro-inflammatory phenotype. This approval establishes a safety precedent for a single iron oxide formulation used for iron replacement; however, it does not extend to magnetic immunotherapy nanoparticles carrying checkpoint inhibitors, ferroptosis inducers, membrane coatings, or constructs requiring external magnetic-field devices. Section 5.3 discusses the regulatory considerations arising from these limitations; they are not reiterated here.

Among the four classes of platforms, magnetic nanoparticle-based systems (MNPs) are unique in offering field-assisted targeting capabilities. Their practical utility is contingent upon factors such as field strength and gradient, tumor depth, vascular flow, particle magnetization, and the constraints imposed by clinical devices [38]. Reported cargoes in MNP-based immunotherapy encompass V-domain Ig suppressor of T cell activation (VISTA) monoclonal antibodies, anti-PD-1 and anti-PD-L1 antibodies, toll-like receptor (TLR) agonists such as poly I:C combined with imiquimod (R837), the ferroptosis inducer sulfasalazine, and hyperthermia used as an immune activator. Fe3O4@TiO2 nanoparticles co-loaded with VISTA checkpoint antibodies have been evaluated in sonodynamic therapy integrated with checkpoint blockade for pancreatic cancer, demonstrating that MNP-mediated reactive oxygen species (ROS) generation can augment checkpoint blockade in a tumor type characterized by poor immunogenicity [39]. A hybrid platform comprising mesoporous silica nanoparticles (MSNs) and iron oxide, consisting of a ferumoxytol-capped ultralarge-pore mesoporous silica construct loaded with anti-PD-L1, is discussed here due to its magnetic imaging properties; however, its performance results from the combined contributions of both the MSN and iron oxide components and should not be interpreted as evidence of MNP efficacy [40].

Platelet membrane-camouflaged iron oxide nanoparticles loaded with sulfasalazine (Fe3O4-SAS@PLT) demonstrated ferroptosis-enhanced anti-PD-1 immunotherapy with M2-to-M1 macrophage repolarization and sustained tumor elimination in a 4T1 metastatic model [41]. These mechanisms, specifically ferroptosis-mediated glutathione (GSH) depletion and macrophage repolarization, should be attributed to this formulation rather than to iron oxide nanoparticles as a class, and tumor-associated macrophage (TAM)-specific GSH depletion and M2-to-M1 repolarization should be cited only where directly measured. Poly I:C-imiquimod MNP plus anti-PD-L1 achieved efficacy against B16-F10 melanoma [42].

Magnetic nanoparticle-mediated hyperthermia using iron oxide nanoparticles with an alternating magnetic field has initiated a registered early-feasibility clinical evaluation for advanced metastatic solid tumors (ClinicalTrials.gov NCT07224464) [11]. Furthermore, an earlier conference abstract documented preliminary safety and feasibility observations for a magnetic hyperthermia approach [43]. This abstract serves as an independent record and is not linked to the data from the registered trial; additionally, clinical evidence supporting the combination with anti-PD-1 or anti-PD-L1 therapies has yet to be established through registered trials.

### 2.4. Covalent Organic Frameworks (COFs)

Many COFs are metal-free crystalline porous organic polymers connected through covalent linkages such as imine, beta-ketoenamine, or triazine bonds. Certain COF nanomedicine designs deliberately incorporate metal centers, metal ions, or nanozyme-like components, so safety and degradation profiles cannot be attributed uniformly across the class [44]. COFs typically exhibits high surface areas (400 to 3000 m^2^/g), adjustable pore geometries, and pi-conjugated backbones that provide light-harvesting and photothermal conversion in photosensitizer-enhanced designs.

#### 2.4.1. Long-Term Biodegradation and Excretion

Biodegradation remains the least advanced aspect of covalent organic framework (COF) nanomedicine and is the most likely factor to influence whether the class proceeds to an investigational new drug application. Covalent linkages are inherently more hydrolytically stable than the coordination bonds that aggregate metal–organic frameworks. This stability, while advantageous for cargo retention, poses challenges for clearance. The in vivo studies reported to date predominantly feature short observation periods, ranging from days to a few weeks. Degradation is generally inferred through indicators such as loss of crystallinity, fluorescence decay, or diminished organ signals, rather than through mass-balance analysis of specific degradation products [44]. Consequently, three critical questions remain unresolved for any COF construct considered for clinical development: the chemical identity and toxicological profile of the framework fragments and released monomers, including the aldehydes and amines for imine-linked frameworks; the quantitative excretion via renal and hepatobiliary pathways; and the organ burden following repeated doses, as opposed to a single administration. Until comprehensive mass-balance data over extended periods, measured in months, are available to address these questions, no COF formulation should be regarded as biodegradable within a regulatory context.

#### 2.4.2. Comparison with Metal–Organic Frameworks

Metal–organic frameworks (MOFs) serve as an informative comparison, as they share attributes such as porosity, crystallinity, and high loading capacity with covalent organic frameworks (COFs), while differing in their bonding. Coordination bonds between metal nodes and organic linkers are susceptible to lability under physiological conditions, leading to comparatively rapid degradation of nanoscale MOFs through ligand displacement and metal ion release. Early biodistribution studies have demonstrated that iron carboxylate MOFs degrade and are effectively cleared, with acceptable levels of acute tolerability [13]. This inherent lability represents both the primary advantage and the main drawback of the MOF class; while it facilitates clearance, it also poses risks of premature cargo release and systemic metal ion burden that require careful evaluation.

Four key differences should directly inform platform selection. degradation: MOF clearance is comparatively rapid and mechanistically well-understood, whereas COF persistence tends to be longer and less characterized. The second is cargo retention: COFs more reliably retain cargo during circulation, which benefits stimulus-responsive designs, while MOFs are more susceptible to burst release upon partial framework collapse. the toxicological profile: the risk associated with MOFs is primarily due to the released metal, whereas the risk for metal-free COFs is mainly attributable to framework persistence and the aromatic monomers produced upon degradation. Consequently, these two classes require distinct toxicological assessments. The fourth is translational maturity: MOF nanomedicines possess a longer track record in vivo within cancer immunotherapy, including chlorin-based nanoscale MOFs that combine photodynamic therapy with checkpoint blockade to achieve local and distant tumor rejection in colorectal models [14]. Conversely, the literature on COF immunotherapy is relatively recent and more limited. Neither class is close to regulatory approval. The practical conclusion is that COFs should be preferred over MOFs when framework stability and designed photophysical properties are mechanistically essential. In such cases, the persistence resulting from COFs must be directly addressed rather than presumed to be harmless.

#### 2.4.3. Targeting and Representative Preclinical Evidence

Selected COF formulations achieve active targeting through hyaluronic acid (CD44-targeted), folic acid (folate receptor-targeted), and Fusobacterium nucleatum bacterial membrane coatings, leveraging intratumoral bacterial tropism to facilitate tumor tissue distribution [45]. GSH-triggered disulfide bond cleavage and hypoxia-responsive framework collapse enable tumor microenvironment (TME)-specific drug release in specific configurations [46].

Selected covalent organic framework (COF) formulations, particularly those integrated with aggregation-induced emission luminogens (AIEgens), have been documented to induce immunogenic cell death (ICD) associated with pyroptosis and ferroptosis in preclinical models. COF-919, an AIEgen-integrated COF, was reported to achieve over 90% tumor growth inhibition and cure rates exceeding 80% in a 4T1 breast cancer model, applicable to both bilateral and rechallenge variants, through reactive oxygen species (ROS)-mediated cleavage of gasdermin E and inactivation of glutathione peroxidase 4 (GPX4) [46]. These findings are specific to this particular formulation, tumor model, treatment regimen, and combination context, and should not be generalized to all COFs as a class. Tumor growth inhibition (TGI) metrics reported across various studies employ different measurement formulas and comparators, rendering direct comparison infeasible. One study indicated an 84.6% inhibition of distant tumors in a bilateral 4T1 model utilizing a bacterial membrane-coated COF (COF-306@FM) [45]. The formation of tertiary lymphoid structures (TLSs) has been observed in selected COF investigations, including the bacterial membrane-coated COF-306@FM [45], as well as in a trigger-inducible TLS-forming COF that elicited cytokine hypersecretion and TLS organization in MC38 and 4MOSC1 tumor models in conjunction with anti-CTLA-4 therapy [47]. These observations are specific to formulations and are not universal characteristics of the four platforms analyzed.

## 3. Comparative Analysis Framework

### Organization of the Evidence Map and Its Limitations

To compile observations across platform families, we synthesized the evidence into two descriptive tables and one qualitative figure. Table 1 consolidates platform-level physicochemical and design characteristics. Table 2 enumerates formulation-specific evidence from the individual studies cited in Section 2.1, Section 2.2, Section 2.3 and Section 2.4, including tumor models, cargo, route and treatment design, reported antitumor outcomes, immune endpoints, and source references. Figure 3 represents this information as a qualitative evidence map using descriptive categories.

This evidence map is hypothesis-generating and does not constitute a validated meta-analysis. Studies were not extracted systematically, and no aggregate numerical score or platform ranking has been assigned, due to substantial differences across studies in tumor models, payloads, routes of administration, doses, schedules, comparators, and immune-endpoint depth. Consequently, the evidence is organized into six descriptive dimensions that maintain these distinctions rather than consolidating them into a single overall score.

The four evidence categories outlined in Figure 3 are established through explicit thresholds applied to the studies referenced for each platform, as detailed in Section 2.1, Section 2.2, Section 2.3 and Section 2.4 and systematically tabulated in Table 2. One of these categories utilizes a numerical decision rule, while the remaining five do not. Specifically, the category for the independent in vivo evidence-based dimension is determined solely by enumerating independent reviewed in vivo formulations listed for each platform in Table 2, employing predefined classifications: “Substantial” for four or more formulations, “Moderate” for three, “Emerging” for two, and Limited for one or none. This dimension is straightforward to reproduce from Table 2, ensuring transparency and consistency.

For dimensions that describe a qualitative characteristic rather than a count, including targeting and delivery strategy, cargo breadth, safety and regulatory precedent, tumor access, and immune activation breadth, the same four classifications are applied descriptively. “Substantial” is reserved for properties confirmed with in vivo evidence in two or more independent, peer-reviewed formulations and at least one directly measured immune endpoint, such as depletion, flow cytometry, or histology. “Moderate” applies where at least one reviewed in vivo formulation shows partial mechanistic characterization. “Emerging” applies where support rests mainly on a single formulation or on predominantly in vitro or design-level evidence. “Limited” applies where the basis is a design-level assertion with no qualifying reviewed in vivo study.

The remaining five dimensions are qualitative judgments assigned by the author, and they are characterized accordingly rather than being reproducible classifications. The criteria employed are not directly comparable: physical distinctiveness of a targeting mechanism, the number of qualifying formulations, the existence of regulatory precedent outside the reviewed set, mechanistic breadth, and the extent of endpoint measurement represent different categories of evidence. The aggregation of these diverse evidence types into a single four-level scale enforces a shared vocabulary on quantities lacking a common metric. Consequently, a second author applying the specified descriptions to the same table might reasonably arrive at different categorizations in several cells. Because these categories are based on a limited, non-systematic collection of reviewed studies and rely on author judgment for five out of six dimensions, they should be regarded as descriptive indicators rather than population-level estimates or a reproducible scoring system. Accordingly, readers should interpret them as the author’s analysis of Table 2 rather than as objective measurements.

A worked example for the count-based dimension is as follows. In Table 2, PLGA lists four independent in vivo formulations [22,23,25,26], MSN lists five, including the hybrid construct [31,32,33,40,48], and COF lists four [45,46,47,49]; thus, each is scored as Substantial, whereas MNP lists three [39,41,42] and is scored as Moderate. The Substantial rating for MSN does not depend on the hybrid MSN–iron oxide construct [40], since excluding it still leaves four qualifying pure-MSN formulations [31,32,33,48] that meet the four-or-more threshold. For the qualitative targeting and delivery strategy dimension, MNP is scored as Substantial because field-assisted targeting and MR guidance are physically distinctive and are reported with in vivo confirmation across the iron oxide and hybrid constructs [39,40,41], while the other platforms, which rely mainly on passive accumulation with selected active-targeting examples, are scored as Moderate. All remaining cells adhere to the same rule specified for the corresponding Table 2 rows, enabling any reader to reproduce a cell by counting or inspecting the qualifying rows for that platform and dimension.

For traceability, the remaining qualitative categories are justified against Table 2 as follows. Cargo classes accommodate the distinct cargo types present in each platform’s reviewed entries. PLGA encompasses tumor lysate, poly I:C, an IDO1 inhibitor, a TLR7/8 agonist, and a photosensitizer with anti-PD-L1, thereby representing four classes and receiving a moderate rating. MSN includes cyclic dinucleotide STING agonists, a manganese-cyclic-dinucleotide complex, and a chemotherapy-plus-checkpoint hybrid, resulting in three classes and a Moderate rating. COF covers AIEgen photosensitizers, a bacterial-membrane biomimetic, a multienzyme nanozyme, and a TLS-inducing design, totaling four mechanistically distinct cargo or coating classes and awarded a substantial rating. MNP rows use a more restricted set focused on iron oxide cores paired with a checkpoint antibody, a ferroptosis inducer, or a TLR agonist, resulting in an Emerging rating that reflects fewer distinct cargo classes than the other platforms.

Safety and regulatory precedents are established by approved products outside of Table 2: PLGA has multiple approved depot formulations and is classified as Substantial [16,17]; MNP has one approved iron oxide product that is not directly applicable to immunotherapy constructs and is classified as Moderate [50,51]; MSN features a hybrid construct incorporating an approved iron oxide capping agent but has no approved MSN product and is classified as Moderate [40]; and COF has no approved products and is categorized as Emerging.

Tumor access in the reviewed models is categorized as Emerging for PLGA due to its reliance primarily on passive accumulation. In contrast, it is rated as Moderate for MSN, MNP, and COF, as their configurations incorporate additional targeting strategies, such as active targeting, field-assisted localization, or bacterial and membrane-directed homing. The breadth of immune activation is evaluated by counting distinct immune mechanisms measured per platform, as detailed in the immune-mechanism column of Table 2: PLGA is classified as Emerging, with a focus on dendritic cell and effector T-cell priming; MSN and MNP are rated as Moderate, primarily centered on STING activation or repolarization with checkpoint enhancement; and COF is deemed Substantial, encompassing pyroptosis, ferroptosis, cGAS-STING activation, and TLS formation across its configurations. These assessments are solely based on the reviewed evidence and are subject to the same limitations as the count-based dimension (Table 1).

**Table 1:** Platform-level physicochemical and design properties of the four selected nanoparticle families. Entries describe general platform characteristics. Particle-size ranges are approximate and formulation-dependent. No composite score or ranking is implied.

Property	PLGA NPs	Mesoporous Silica NPs	Magnetic NPs	Covalent Organic Frameworks
Composition	Biodegradable polyester (lactide/glycolide copolymer)	Amorphous silica framework; biodegradable variants (bMSNs)	Iron oxide cores (Fe_3_O_4_, gamma-Fe_2_O_3_); superparamagnetic behavior only below a critical core diameter of approximately 20–30 nm [37]	Crystalline porous organic polymer; some designs include metal centers
Delivery/release strategy	Passive EPR; active targeting (mannose, RGD, aptamer) in specific designs	Passive EPR; surface functionalization; pH/degradation-triggered release	Field-assisted targeting potential; passive uptake; AMF/MHT at 42–46 degrees C	Active targeting (HA, folate, bacterial membrane); hypoxia-/GSH-responsive release in specific designs
Approximate particle size in reviewed formulations (illustrative, not platform-defining)	~100–300 nm [22,24]	~50–200 nm [28,48]	Core diameters span the superparamagnetic and stable single-domain regimes; cores below ~20–30 nm are superparamagnetic, larger cores are single-domain ferrimagnets, and coated or hybrid constructs have substantially larger hydrodynamic diameters [36,37,39]	~50–200 nm [44,46]
Representative cargo classes	Metabolic inhibitors (1-MT), TLR agonists (R848, poly I:C), ICIs, antigens, siRNA	CDN STING agonists; Mn^2+^ complexes; ICIs; antigen plus CpG; DMXAA (murine only)	ICIs (VISTA mAb, anti-PD-1), TLR agonists, ferroptosis inducers, MHT modality	Photosensitizers (AIEgens), pyroptosis/ferroptosis inducers, ICIs, chemotherapeutic prodrugs in specific designs
Principal formulation and manufacturing constraint	Antigen denaturation during encapsulation, acidic degradation microclimate, burst release, and absence of any marketed PLGA nanomedicine owing to scale-up barriers [18,19,20]	Formulation-specific degradation kinetics spanning hours to months; silanol-dependent hemolysis and complement activation [29,30]	Field dosimetry, heating heterogeneity, total iron burden on repeat dosing, and device–drug combination regulation [38]	Solvothermal synthesis not cGMP-compatible; batch-to-batch crystallinity control; undefined degradation products [44]
Principal safety/regulatory context	Polymer precedent from approved depot microsphere products; nanoparticle immunotherapy formulations require individual IND packages	Amorphous and biodegradable variants generally tolerated in the reviewed studies; dose-, morphology-, and surface-dependent silica effects possible; first-in-human precedent limited to ultrasmall renally cleared silica imaging particles [12]	Ferumoxytol provides an iron-replacement precedent for one formulation only; immunotherapy MNPs require separate characterization	Limited long-term in vivo biodegradation, excretion, and repeat-dose safety data; metal-containing variants add complexity

**Table 2:** Formulation-level evidence from the representative studies cited in Section 2.1, Section 2.2, Section 2.3 and Section 2.4, compiled to allow comparison of study design across platforms. Each row records the administration route and treatment design, the comparator arm, the antitumor outcome with the qualitative endpoint described in the source, the basis on which tumor growth inhibition or response was defined, the survival outcome, toxicity observations, and whether the principal immune mechanism was directly measured or inferred. Numerical doses and dosing schedules were not transcribed and the column records route, targeting approach, and treatment combination only. Exact administered doses, full dosing schedules, specific endpoint days, and source-specific quantitative TGI formulas remain in the cited primary publications. “nr” indicates the field was not explicitly reported in the cited source. Reported TGI and outcome values use different formulas, models, schedules, and comparators across studies and are not directly comparable.

Platform	Formulation/Cargo	Tumor Model	Route and Treatment Design	Comparator Arm	Antitumor Outcome (Endpoint)	TGI Definition/Basis	Survival	Toxicity Observations	Immune Mechanism (Measured vs. Inferred)	Ref.
PLGA	Mannosylated PLGA + tumor cell lysate + poly I:C	Syngeneic 4T1 murine breast cancer	Subcutaneous vaccination; repeated prime-boost dosing	Untreated and non-targeted PLGA controls	Reduced primary tumor burden and lung metastases	Tumor size/burden reduction vs. control (volume-based)	2–3× survival improvement	No overt toxicity reported	Cellular immunity (lymphocyte proliferation, DTH, CD107a, increased IFN-gamma) directly measured; CD8 causality inferred, not depletion-tested	[22]
PLGA	RGD-targeted lipid/PLGA + 1-MT (IDO1 inhibitor) + Ce6 + DPPA (PD-L1 peptide inhibitor)	CT26 colorectal (murine)	Intravenous; RGD-targeted; photodynamic activation	Free drug; non-targeted nanocomplex	Tumor-growth inhibition with photoimmunotherapy	Tumor volume reduction vs. control (volume-based)	Immune-memory prevention of metastasis reported	No overt toxicity reported	DC maturation, IDO inhibition, increased tumor-infiltrating CTL directly measured	[23]
PLGA	Mannosylated PLGA + R848 (TLR7/8 agonist)	TAM-rich murine tumor model	Systemic; mannose-targeted to TAMs	Free R848; non-targeted NP	TAM reprogramming and tumor control	Tumor volume reduction vs. control (volume-based)	nr	Reduced systemic cytokine toxicity vs. free R848	Increased TNF-alpha/IL-12, M2-marker suppression directly measured	[26]
PLGA	M1 macrophage membrane-coated PLGA + IR780 + catalase (SDT) + anti-PD-L1	4T1 metastatic (murine)	Intravenous; ultrasound activation; systemic anti-PD-L1	PLGA without anti-PD-L1; PBS	Effective metastasis blockade (no abscopal endpoint reported)	Metastasis/tumor control vs. control (volume-based)	nr	No overt toxicity reported	ICD (CRT exposure) and DC activation directly measured	[25]
MSN	c-di-GMP-loaded MSN (STING agonist)	4T1 breast cancer (murine)	Intratumoral; CDN-loaded MSN	Free c-di-GMP; empty MSN	Enhanced antitumor immunity vs. free agonist	Tumor volume reduction vs. free CDN (volume-based)	nr	nr	Type I IFN and DC activation directly measured	[48]
MSN	bMSN + STING agonist (CDN)	B16 murine melanoma	Intratumoral/peritumoral; CDN-loaded biodegradable MSN	Free CDN; empty bMSN	Prolonged survival; antitumor efficacy	Tumor volume reduction vs. free CDN (volume-based)	Prolonged survival reported	nr	Type I IFN, DC maturation, CD8 cross-priming measured; CD8 depletion reported	[31]
MSN	CDA-Mn@bMSN (CDN + Mn^2+^)	Large established orthotopic B16 melanoma	Systemic/intratumoral; CDN + Mn co-load; +anti-PD-1/PD-L1	CDN alone; bMSN alone; ICB alone	Strong regression of large tumors; ICB synergy	Tumor regression vs. monotherapy (volume-based)	Improved survival reported	nr	STING amplification and systemic activation measured; Mn contribution inferred	[32]
MSN	Mitochondria-targeted Mn^2+^ MSN + anti-PD-L1	4T1 TNBC (poorly immunogenic)	Systemic; +anti-PD-L1	Anti-PD-L1 alone; vehicle	Tumor-growth suppression	Tumor volume vs. control (volume-based)	nr	nr	cGAS-STING activation and anti-PD-L1 sensitization directly measured	[33]
MSN-Fe (hybrid)	Ferumoxytol-capped ultralarge-pore MSN + anti-PD-L1 (after cabazitaxel)	TRAMP-C1 prostate cancer (murine)	Intratumoral; MR image-guided; sequential after cabazitaxel chemotherapy	Systemic anti-PD-L1 of same dose; chemotherapy alone	Tumor regression superior to systemic anti-PD-L1	Tumor volume reduction vs. systemic ICB (volume-based)	nr	nr	Increased CD8+ T cell infiltration and reduced Treg directly measured; no abscopal endpoint	[40]
MNP	Fe_3_O_4_-SAS@PLT (sulfasalazine)	4T1 metastatic (murine)	Intravenous; platelet-membrane camouflaged; +anti-PD-1	Anti-PD-1 alone; bare NP	Continuous tumor elimination	Tumor volume/weight reduction vs. control (volume-based)	nr	nr	Ferroptosis and M2-to-M1 repolarization measured; ferroptosis-to-repolarization link inferred	[41]
MNP	Poly I:C-imiquimod MNP + anti-PD-L1	B16-F10 melanoma (murine)	Intratumoral/systemic; TLR agonist co-load; +anti-PD-L1	Free agonists; anti-PD-L1 alone	Antitumor efficacy	Tumor control vs. control (volume-based)	nr	nr	Innate activation and checkpoint synergy measured; mechanism partly inferred	[42]
MNP	Fe_3_O_4_@TiO_2_ + VISTA mAb (SDT)	Pancreatic cancer (poorly immunogenic, murine)	Intratumoral/systemic; ultrasound; +VISTA mAb	SDT alone; VISTA mAb alone	ROS-augmented checkpoint blockade	Tumor control vs. control (volume-based)	nr	nr	VISTA blockade and ROS-driven ICD directly measured	[39]
COF	COF-919 (AIEgen-integrated) + anti-PD-1	4T1 breast cancer (BALB/c, *n* = 5); bilateral and rechallenge models	Intratumoral/systemic; NIR phototherapy; +anti-PD-1	Non-AIEgen COF; PBS; untreated	More than 90% TGI; more than 80% cure rate; primary and distant tumor control	Volume-based TGI vs. untreated control	Improved percent survival (log-rank); rechallenge resistance	No overt toxicity reported	Pyroptosis (GSDME) and ferroptosis (GPX4) directly measured; cell-non-autonomous STING inferred	[46]
COF	COF-306@FM (Fusobacterium nucleatum membrane-coated) + anti-PD-L1	Bilateral 4T1 breast cancer (murine)	Intratumoral primary + systemic anti-PD-L1; NIR phototherapy	COF without membrane; anti-PD-L1 alone	84.6% distant-tumor inhibition; suppressed metastasis/recurrence	Distant-tumor inhibition vs. control (volume-based)	Reduced recurrence/metastasis reported	nr	TLS formation and CD8+/B cell infiltration measured by histology; causal requirement inferred	[45]
COF	Trigger-inducible TLS-forming AIE COF (phototherapy) + anti-CTLA-4	MC38 colorectal and 4MOSC1 oral squamous (murine)	Intratumoral; phototherapy; +anti-CTLA-4	COF alone; anti-CTLA-4 alone; untreated	Eradication of primary and distant tumors with anti-CTLA-4 synergy	Tumor volume reduction vs. control (volume-based)	Improved survival reported	nr	TLS formation and cytokine hypersecretion directly measured by single-cell sequencing and histology; T/B cell maturation measured	[47]
COF	PEG-CuP-COF@dSt (multienzyme nanozyme + tumor-targeting Salmonella; sonodynamic)	Murine tumor model (with bone-metastasis assessment)	Systemic; bacteria-mediated targeting; ultrasound activation	Free COF; bacteria alone; untreated	Tumor-growth inhibition via pyroptosis	Tumor volume reduction vs. control (volume-based)	Immune-memory prevention of bone metastasis reported	nr	Caspase-3/GSDME pyroptosis and ROS generation directly measured; immune-memory contribution inferred	[49]

Abbreviations (Table 1 and Table 2): AIEgen, aggregation-induced emission luminogen; AMF, alternating magnetic field; bMSN, biodegradable mesoporous silica nanoparticle; CDA, cyclic di-AMP analog; CDN, cyclic dinucleotide; cGAS, cyclic GMP-AMP synthase; cGMP, current good manufacturing practice; COF, covalent organic framework; CRT, calreticulin; DAMP, damage-associated molecular pattern; DC, dendritic cell; DTH, delayed-type hypersensitivity; EPR, enhanced permeability and retention; GPX4, glutathione peroxidase 4; GSDME, gasdermin E; GSH, glutathione; HA, hyaluronic acid; ICB, immune checkpoint blockade; ICD, immunogenic cell death; ICI, immune checkpoint inhibitor; IFN, interferon; IND, investigational new drug; MDSC, myeloid-derived suppressor cell; MHT, magnetic hyperthermia; MNP, magnetic nanoparticle; MRI, magnetic resonance imaging; MSN, mesoporous silica nanoparticle; 1-MT, 1-methyl-tryptophan; nr, not reported; PD-1, programmed cell death protein 1; PD-L1, programmed death-ligand 1; PLGA, poly(lactic-co-glycolic acid); ROS, reactive oxygen species; SAS, sulfasalazine; SDT, sonodynamic therapy; SPION, superparamagnetic iron oxide nanoparticle; STING, stimulator of interferon genes; TAM, tumor-associated macrophage; TGI, tumor growth inhibition; TLR, Toll-like receptor; TLS, tertiary lymphoid structure; TNBC, triple-negative breast cancer; Treg, regulatory T cell; VISTA, V-domain Ig suppressor of T cell activation.

## 4. Immunological Mechanisms Underpinning Platform Performance

### 4.1. cGAS-STING Pathway Activation

The cGAS-STING pathway is a fundamental element of innate immune surveillance concerning cytosolic DNA. cGAS recognizes double-stranded DNA and synthesizes cGAMP, which activates STING, recruits TBK1 and IRF3, and promotes transcription of type I interferons and pro-inflammatory cytokines. This process results in dendritic cell (DC) maturation and facilitates cross-priming of CD8+ T cells [31]. The extent of activation varies by formulation and species; therefore, the strategies for engaging STING outlined below should not be reduced to a single comparison of pathway potency.

Biodegradable and cationically modified MSN formulations are well suited for the delivery of STING agonists, as pore confinement safeguards cyclic dinucleotides against enzymatic degradation. Moreover, pH-triggered dissolution of bMSN facilitates cytosolic delivery in proximity to cGAS [32,48]. In configurations such as CDA-Mn@bMSN, manganese enhances STING signaling by sensitizing cGAS and providing endogenous mitochondrial DNA through mitochondrial membrane permeabilization [32]. The effectiveness of these formulations depends on pore architecture, surface charge, degradation kinetics, and formulation-specific endosomal escape efficiency [31,32,48].

In specific AIEgen-integrated COF formulations, indirect cGAS-STING activation has been documented through pyroptosis-mediated cytosolic mtDNA release following caspase-3 and gasdermin E cleavage [46]. This cell non-autonomous cGAS activation is a formulation-specific finding for COF-919 and should not be generalized to COF nanomedicines as a class. Because CDN-loaded bMSNs, manganese-amplified STING systems, and COF-induced mtDNA release are mechanistically distinct, their relative potency cannot be ranked from the heterogeneous studies currently available.

### 4.2. Immunogenic Cell Death (ICD) Induction

ICD is characterized by the emission of damage-associated molecular patterns (DAMPs), including calreticulin (CRT) surface translocation, ATP secretion, and high-mobility group box 1 (HMGB1) release, which transform immunologically silent cell death into a dendritic cell (DC)-activating and T cell-priming event [25].

In sonodynamic PLGA formulations, the IR780 photosensitizer generates reactive oxygen species (ROS) upon ultrasound activation, thereby inducing endoplasmic reticulum stress and calreticulin (CRT) translocation in tumor cells [25]. In iron oxide magnetic nanoparticle (MNP) formulations, alternating magnetic field (AMF)-driven hyperthermia activates the unfolded protein response and facilitates CRT exposure [36], while simultaneous iron-catalyzed lipid peroxidation induces ferroptosis and enhances damage-associated molecular pattern (DAMP) release [41]. Additionally, in selected formulations integrating aggregation-induced emission (AIE)gens and nanozyme-like covalent organic frameworks (COFs), concurrent pyroptosis, characterized by gasdermin E pore formation and the release of interleukin-1beta and interleukin-18, along with ferroptosis, marked by glutathione peroxidase 4 (GPX4) inactivation and lipid hydroperoxide accumulation, has been reported to generate a broader spectrum of DAMPs than single-mechanism immunogenic cell death (ICD) inducers [46,49]. These findings are specific to particular formulations, and because the studies use different tumor models without a direct comparison, the evidence does not support a definitive ranking of ICD potency across platforms.

### 4.3. Tumor Microenvironment Remodeling

The immunosuppressive tumor microenvironment (TME), comprising regulatory T cells (Tregs), myeloid-derived suppressor cells (MDSCs), M2-polarized tumor-associated macrophages (TAMs), and PD-L1 upregulation, poses a primary challenge to cancer immunotherapy. As illustrated in Figure 2, each of the four platform categories has been documented to address this challenge through various mechanisms within specific formulation investigations.

In mannosylated PLGA formulations, R848 activates TLR7/8 in macrophages expressing mannose receptors, raising tumor necrosis factor-alpha and IL-12 while suppressing M2-associated markers [26]. Co-delivery of the IDO1 inhibitor 1-MT within PLGA-based nanocomplexes inhibits the tryptophan-kynurenine pathway and relieves Treg-mediated suppression of effector T cells [23].

Iron oxide magnetic nanoparticle formulations, particularly platelet membrane-camouflaged Fe_3_O_4_-SAS, have demonstrated that ferroptosis-mediated inhibition of SLC7A11 contributes to synergistic effects with anti-PD-1 therapy and facilitates macrophage reprogramming in a 4T1 model [41].

Magnetic hyperthermia is frequently suggested as a method for loosening dense desmoplastic stroma; however, this proposition warrants correction from a thermal perspective. Therapeutic magnetic hyperthermia typically operates within a temperature range of approximately 42 to 46 degrees Celsius. Temperatures exceeding roughly 50 to 60 degrees Celsius are classified as thermal ablation rather than hyperthermia [36]. Collagen denaturation does not occur significantly within this range. Direct measurements indicate that collagen tissue exhibits only about a 2% loss of structural signal after two hours at 50 degrees Celsius and approximately 25% after two hours at 55 degrees Celsius, while substantial denaturation occurs within seconds to minutes only at temperatures between 60 and 70 degrees Celsius [52]. Consequently, attributing stromal loosening during magnetic hyperthermia to collagen denaturation is unsupported at therapeutic field settings. Any observed enhancement of nanoparticle penetration or T cell infiltration under hyperthermic conditions should thus be ascribed to mechanisms operative within the 42 to 46 degrees Celsius range. These mechanisms primarily include increased vascular perfusion and permeability, altered interstitial fluid pressure, heat shock protein expression, and changes in endothelial and cellular adhesion. Collagen denaturation becomes a plausible factor exclusively in intentionally ablative protocols, which possess a different safety profile and should be considered as ablation rather than hyperthermia.

Biodegradable MSN formulations deliver STING agonists to induce type I interferon production, which can restrain MDSC expansion and Treg recruitment, thereby transforming immunologically cold tumors into hot tumors in specific models [31,32,33,48]. Reports indicating that sorafenib co-delivery inhibits the production of immunosuppressive cytokines by cancer-associated fibroblasts are not substantiated by the formulations cited in this section; therefore, such assertions are not endorsed herein.

In certain COF formulations, hypoxia-responsive generation of reactive oxygen species (ROS) results in the depletion of intratumoral glutathione (GSH) and prompts pyroptosis-mediated release of chemokines that promote tertiary lymphoid structure (TLS) formation, namely CXCL13, CCL19, and CCL21. In COF-mediated phototherapy models, TLS formation creates organized ectopic lymphoid structures that facilitate local antigen presentation and support sustained antitumor immune surveillance. This mechanism exhibits dependency on the specific formulation.

### 4.4. Immune Checkpoint Blockade Synergy

A consistent observation across all four platform categories is that nanoparticle-mediated immune activation can synergize with PD-1/PD-L1 or CTLA-4 blockade by transforming cold tumors, characterized by insufficient T cell infiltration, into hot tumors that are more responsive to immune checkpoint blockade (ICB) [25,31]. In the studies examined, researchers typically administer the checkpoint antibody systemically as a separate agent rather than physically incorporating it into the nanoparticle, except in cases where a construct explicitly co-encapsulates the antibody. This distinction is significant because the term “platform plus ICB” does not imply the use of a single, combined construct.

Regarding PLGA, an M1 macrophage membrane-coated construct co-loading IR780 and catalase was utilized to mediate sonodynamic ICD, which, in conjunction with systemic anti-PD-L1 therapy, inhibited metastasis in the 4T1 model [25]. For MSN carriers, the delivery of STING agonists was successfully established in bMSN formulations [31], and in manganese-enhanced STING formulations, the transient elevation of PD-L1 was mitigated through combination with anti-PD-1 or anti-PD-L1 agents [32,33]. Regarding the hybrid MSN–iron oxide platform, sequential MR image-guided local delivery of anti-PD-L1 following cabazitaxel chemotherapy resulted in tumor regression that surpassed that achieved with systemic anti-PD-L1 in a TRAMP-C1 prostate cancer model [40]. As this construct is a hybrid rather than a pure iron oxide nanoparticle, the observed checkpoint-synergy outcome provides evidence for the behavior of hybrid MSN–iron oxide particles, rather than pure magnetic nanoparticles. For COFs, specific preclinical models showed substantial eradication or inhibition of primary and distant tumors when COF formulations were combined with checkpoint blockade, attributed to dual induction of ICD and enhanced effector T cell responses [45,46]. These findings reflect the individual design of formulations and tumor models, and such combination therapies should remain a priority in preclinical research until their safety profiles are fully established.

### 4.5. Strength of Mechanistic Evidence

The strength of evidence supporting the immune mechanisms invoked across these studies varies, and mechanisms that have been directly tested are distinguished here from those that are inferred. The bMSN-STING melanoma study demonstrated CD8 dependence by showing that antibody-mediated CD8 depletion abolished protection [31]. In the mannosylated PLGA vaccine study, tumor-specific cellular immunity was directly assessed through lymphocyte proliferation, delayed-type hypersensitivity, CD107a expression, and interferon-gamma response, with a two- to three-fold survival increase, but CD8 dependence was not tested by depletion, so the causal role of CD8 T cells is inferred rather than confirmed [22]. Macrophage M2-to-M1 repolarization was directly evaluated by phenotyping in the platelet membrane-camouflaged Fe_3_O_4_-SAS study, but the upstream contribution of ferroptosis to that repolarization was inferred from pathway markers rather than demonstrated by a ferroptosis-specific rescue [41].

Reductions in Treg and MDSC populations attributed to STING-driven type I interferon were measured by flow cytometry in CDN-loaded bMSN studies, yet the causal link to MDSC suppression was inferred from correlation with type I interferon output [31,48]. In the hybrid construct, increased CD8+ T cell infiltration and decreased Treg proportions were directly observed in the prostate model, but no bilateral or abscopal endpoints were reported, so distant-site activity is not asserted [40].

TLS formation in the bacterial membrane-coated COF study was directly visualized through histology and immunostaining; however, its necessity for the antitumor effect was inferred rather than definitively confirmed by TLS ablation [45]. Abscopal-type distant-tumor growth inhibition was directly observed in the bilateral COF-306@FM model, whereas attributing that effect specifically to checkpoint blockade was inferred from comparative analysis across treatment groups [45]. When a mechanism is inferred rather than definitively confirmed by depletion, it should be regarded as hypothesis-generating.

## 5. Clinical Translation Considerations

The clinical translation trajectory of each platform is governed by formulation-specific factors, namely exact formulation, route, sterility, endotoxin, residual solvent, release profile, immunotoxicity, biodistribution, clearance, repeat-dose toxicology, and manufacturing reproducibility, rather than by platform identity alone [28]. Platform classes differ in translational maturity, and the evidence descriptors in Table 1 and Table 2 reflect those differences. Clinical-stage descriptors should be read as descriptions of current development stage, not as rankings of platform importance.

### 5.1. PLGA Nanoparticles

PLGA possesses the most substantial translational foundation among the four reviewed classes concerning polymer precedent. Approved long-acting injectable PLGA-containing depot microsphere products such as Lupron Depot and Risperdal Consta serve as exemplars of regulatory and manufacturing precedent for the polymer as a depot excipient [16,17]. This precedent is distinct from the regulatory and scientific standards necessary for PLGA nanoparticles utilized in immunotherapy. Each such formulation represents a unique product requiring a product-specific Investigational New Drug (IND) submission that addresses aspects such as polymer molecular weight, lactide-to-glycolide ratio, end-group chemistry, residual solvents, encapsulation efficiency, antigen integrity, release profile, sterility, endotoxin levels, and safety in conjunction with immune checkpoint blockade (ICB) agents. The approval of depot products does not mitigate the risks associated with nanoparticle vaccines or immunotherapy formulations [27]. The manufacturing and antigen-stability considerations elaborated in Section 2.1.1 and Section 2.1.2 constitute the critical path for these programs.

### 5.2. Mesoporous Silica Nanoparticles

bMSN formulations offer a compelling preclinical rationale for the delivery of cytosolic STING agonists. Translation into clinical applications necessitates validation of scalable sterile manufacturing processes, reproducible loading and release of CDNs, evaluation of complement activation, hemolysis, and cytokine-release risks, batch consistency regarding pore and surface characteristics, demonstration of biodegradation and silicic acid excretion, as well as IND-enabling repeat-dose toxicology studies in relevant species. Platforms based on bMSN-STING agonists are suitable candidates for IND-enabling development; however, a definitive clinical timeline cannot be established until these studies are completed. The clinical experience with free CDN STING agonists [34,35] delineates the specific efficacy hypothesis that any bMSN-STING program must be designed to test, namely, that enhanced intratumoral retention and cytosolic delivery translate into clinical activity not achieved by free agonists.

### 5.3. Magnetic Nanoparticles

The precedent set by ferumoxytol, as described in Section 2.3, establishes clinical safety for a single iron oxide formulation employed for iron replacement therapy; however, it does not extend to magnetic immunotherapy nanoparticles that carry checkpoint inhibitors, ferroptosis inducers, membrane coatings, or constructs necessitating external-field devices [50]. Magnetic nanoparticle (MNP) immunotherapy programs must independently address various considerations, including iron metabolism and overall iron burden on repeated dosing, reticuloendothelial accumulation, oxidative stress, MRI quantification, performance of field-assisted targeting, safety of alternating magnetic fields (AMFs), specific absorption rate, heterogeneity of heating, and regulation of device–drug combinations. The latter is particularly noteworthy: constructs requiring an external field applicator constitute a combination product, with development obligations on two regulatory pathways concurrently, and field dosimetry becomes a critical quality attribute akin to particle size. Progress has been made in magnetic nanoparticle-mediated hyperthermia, which has advanced into registered early-feasibility clinical evaluations for patients with advanced metastatic solid tumors [11], supplemented by an earlier conference abstract reporting initial safety and feasibility observations [43]. These instances are distinct, and supporting clinical evidence for combination therapies involving anti-PD-1 or anti-PD-L1 agents remains to be substantiated through registered clinical trials.

### 5.4. Covalent Organic Frameworks

COF immunotherapy platforms remain in the preclinical stage, and their clinical deployment timing cannot be precisely estimated. Essential steps include establishing scalable cGMP-compatible synthesis, given that current solvothermal methods are not compliant with cGMP standards; demonstrating reproducibility in batch-to-batch crystallinity and particle size; characterizing the identity and toxicity of degradation products; measuring biodistribution and excretion profiles; assessing hemocompatibility and immunogenicity; and establishing safety for repeated dosing in relevant animal models. For bacterial membrane-coated COF designs, additional evaluation is required to address the immunogenic and microbiological risks associated with pathogen-derived membrane coatings. Safety and degradation profiles cannot be generalized across the entire COF class, as metal-containing and metal-free designs exhibit distinct toxicological characteristics [44]. Therefore, COFs are regarded here as a preclinical research priority rather than as candidates suitable for IND-oriented development.

## 6. Conflicting Evidence, Reproducibility, and Clinical Attrition

### 6.1. Conflicting Findings and Contradictory Evidence

Three active disputes directly affect how the evidence in this review should be weighted, and none is settled.

The primary concern relates to the extent of nanoparticle dosage that effectively reaches a tumor. A comprehensive review of literature spanning ten years reported a median delivery efficiency of 0.7% of the injected dose, a statistic that has fostered a decade of skepticism regarding nanoparticle targeting [53]. A reexamination of the same foundational datasets employing conventional pharmacokinetic endpoints revealed that the percentage of injected dose within the tumor demonstrated poor correlation with the area-under-curve ratio between tumor and blood, thereby indicating that the metric itself, rather than nanoparticle delivery, was problematic [54]. Both analyses are methodologically justifiable and support contrasting interpretations concerning whether nanoparticle delivery is inherently inefficient or merely inadequately characterized. The practical implication for the reviewed studies is that biodistribution should be documented using explicit pharmacokinetic parameters rather than relying solely on single-timepoint percentage of injected-dose values.

The secondary dispute pertains to the mechanism facilitating tumor entry. The Enhanced Permeability and Retention (EPR) effect, recognized as passive extravasation through inter-endothelial gaps, has served as the fundamental design principle for the majority of passively targeted nanoparticles, including most PLGA and MSN constructs examined herein. Empirical evidence from direct imaging and modeling across four murine models and three human tumor types suggests that inter-endothelial gaps are not the principal pathway; rather, up to 97% of nanoparticles infiltrate tumors via an active transcytotic process traversing endothelial cells [55]. If this finding is broadly applicable, then size-based design optimizations aimed at exploiting fenestrations may be misguided, and the actual focus should be on receptors and vesicular pathways mediating transcytosis. This issue remains unresolved, and the studies listed in Table 2 that reference the EPR effect do so without directly examining the transportation route.

The third dispute pertains to whether preclinical checkpoint enhancement should be regarded as a formulation property or a model property. All combination outcomes presented in Table 2 were derived from a syngeneic murine model, predominantly using 4T1 or B16 cells, which were injected either subcutaneously or orthotopically into immunocompetent inbred hosts characterized by a high mutational burden relative to most human tumors and a condensed disease progression. These models tend to systematically overestimate the responses to immunotherapy. Additionally, when two studies conducted within a single platform report varying degrees of checkpoint synergy, the differences in the model, schedule, and comparator are sufficiently substantial that they cannot be ascribed to the formulation. Consequently, no ranking is provided in this review.

### 6.2. Platform-Specific Conflicts and the Independence of the Evidence Base

The disputes outlined above are pervasive across the field. Whether the four platforms individually exhibit internally conflicting results is an independent matter, and the accurate answer varies depending on the platform.

Regarding PLGA, no genuine conflict exists within the reviewed set; however, the underlying reason is more instructive than reassuring. The two mannosylated constructs [22,26] share a targeting ligand but serve different functions: one primes antigen-presenting cells with tumor lysate and poly I:C, while the other reprograms tumor-associated macrophages with R848. These studies involve different tumor models and distinct routes of administration. Consequently, they are not replicates and cannot corroborate or contradict each other. The PLGA literature examined here is too limited to establish reproducibility, and the absence of contradiction should not be interpreted as evidence of agreement.

There exists a genuine conflict within the reviewed set concerning MSNs, as well as a disparity between preclinical and clinical evidence. Within the dataset, biodegradability is regarded as essential in the bMSN study, where pH-triggered framework dissolution is identified as the mechanism facilitating cytosolic cyclic dinucleotide release [31]. Conversely, comparable enhancement over free agonist is reported for a conventional non-degradable MCM-type carrier [48]. The potential explanatory variables include framework degradability, tumor model (B16 melanoma versus 4T1), and the comparator used to measure enhancement; however, the reviewed data do not distinguish among these factors. The more substantial conflict concerns platform and payload: preclinical MSN-STING literature reports significant activity, whereas free cyclic dinucleotide agonists yielded only one partial response in 47 patients and showed minimal activity in 106 patients when combined with PD-1 blockade [34,35]. This contradiction necessitates resolution rather than being a mere difference in emphasis.

Regarding MNPS, the reviewed compilation presents a direct contradiction concerning whether bare iron oxide is therapeutically active. Ferumoxytol alone has been reported to inhibit tumor growth through pro-inflammatory macrophage polarization, thereby rendering the bare particle an active agent [56]. Conversely, two of the three reviewed MNP immunotherapy formulations employ bare nanoparticle arms as inactive controls, against which the loaded construct is assessed [41,42]. These positions cannot both be correct at the same iron dose. The variables plausibly responsible include the administered iron dose, dosing schedule, particle coating and its influence on macrophage uptake, the tumor model utilized, and the timing of assessment relative to macrophage recruitment. Until these discrepancies are resolved, the efficacy attributed to a loaded MNP construct over a bare-particle control may be underestimated, or the contribution of the bare particle may remain unaccounted for.

Regarding COFs, no conflicts of interest can be identified because the evidence base lacks independent replication. Three of the four reviewed COF formulations, namely COF-919 [46], the bacterial membrane-coated COF-306@FM [45], and the trigger-inducible tertiary lymphoid structure-forming COF [47], share a senior author and originate from a single research program. Consequently, the apparent mechanistic breadth of the COF evidence presented in Figure 3 reflects the productivity and scope of a single research group rather than corroborative findings from multiple independent laboratories. This observation does not diminish the technical robustness of that work but highlights a limitation in interpreting the overall evidence. Such details are not readily apparent from the reference list without examining authorship information. Furthermore, no cross-laboratory reproduction of any COF-related results reviewed has been identified.

### 6.3. Reproducibility and Reporting

Reproducibility in this field is constrained less by the quality of experimental procedures than by reporting practices. The minimum information standards for bio-nano experimental literature specify the necessary material characterization, biological characterization, and protocol details required for a study to be independently interpretable. Adoption of these standards remains partial [57]. The repercussions of this are evident in Table 2. The “nr” entries in the survival and toxicity columns do not necessarily represent gaps in the underlying experiments in every instance but rather gaps in the reported data. Comparator arms vary across studies in significant ways that affect interpretation, as a formulation compared against a free drug and a formulation compared against an empty carrier address different questions. TGI is usually reported without a specified formula in most sources, meaning that a value of 90% in one publication and 84.6% in another cannot necessarily be compared on a common scale.

Particle characterization is generally reported as a mean hydrodynamic diameter and a polydispersity index, measured in buffer rather than in serum, thereby excluding the protein corona that determines biological identity in vivo. Independent replication of formulation-specific efficacy results across laboratories is uncommon in this literature; moreover, this review found no instance among the examined formulations in which one group successfully replicated another group’s construct and outcome. As outlined in Section 6.2, part of the COF evidence base originates from a single research program. Assertions of platform superiority should therefore be interpreted in light of this limitation, rather than based solely on the number of supporting citations.

### 6.4. Why Promising Preclinical Systems Fail in Clinical Trials

The attrition figures provided in Section 1.2 quantify the issue: approximately 48% success rate in completed Phase II trials and approximately 14% in Phase III trials, with failures predominantly attributable to insufficient efficacy rather than toxicity [8]. The Phase III comparison of the paclitaxel-loaded polymeric micelle NK105 against conventional paclitaxel in metastatic breast cancer serves as an illustrative case, as NK105 met its safety expectations and demonstrated reduced peripheral neuropathy; however, it failed to establish non-inferiority for progression-free survival, with median survival durations of 8.4 months versus 8.5 months [58]. A nanoparticle that enhances tolerability without improving efficacy fails to fulfill a clinical need if the comparator remains already tolerable.

Six recurring causes account for most of this attrition, each evident in the preclinical literature reviewed herein. The first is the delivery gap: tumor accumulation in humans is lower and significantly more variable among patients and lesions than in inbred murine models; furthermore, human EPR heterogeneity has no equivalent in a subcutaneous 4T1 tumor [53,55]. The econd is model mismatch: murine syngeneic tumors tend to be fast-growing, homogeneous, and highly immunogenic relative to human disease, and they lack the stromal density and treatment-refractory characteristics of the metastatic populations enrolled in early-phase trials. The third is the comparator problem: preclinical comparators are usually untreated controls or free drug, whereas the clinical comparator is an effective standard of care; hence, a preclinical effect size does not necessarily predict a clinical benefit. The fourth is manufacturing: the material that succeeds in preclinical stages is frequently not the same as that which can be produced under cGMP conditions at scale; process modifications affecting size distribution, surface chemistry, or loading can alter biological behavior [20]. The fifth is mechanism attribution: as discussed in Section 4.5, many mechanistic claims are inferred rather than directly tested through depletion experiments, leading to the possibility that a program advances based on a mechanism that does not function as presumed. The sixth is pharmacological insufficiency of the payload itself, exemplified by the lessons learned in STING agonist trials, where limitations arose from intratumoral retention and cytosolic access rather than target engagement [34,35].

The implications for the four platforms examined are specific rather than broad. Development of formulation programs should ensure that the mechanism claimed is the mechanism tested, that biodistribution is quantified within a model exhibiting realistic stromal architecture, that the manufacturing process is established prior to IND-enabling preclinical studies, and that the comparator accurately reflects the standard of care to which the product will ultimately be subjected.

## 7. Research Priorities and Translational Gaps

Based on the evidence synthesized in this critical comparative review, the following research priorities and translational gaps are identified. Clinical prioritization should rest on formulation-specific manufacturability, safety, route feasibility, and biomarker-defined patient populations rather than on platform class. The examples below are illustrative settings in which existing immunotherapy infrastructure could support combination studies, not evidence-based disease rankings.

### 7.1. PLGA: Near-Term Development Priority

Among the examined platforms, PLGA-based personalized nanoparticle vaccines possess the most advanced precedent in polymer manufacturing and have demonstrated compatibility in co-delivering antigens and adjuvants. Clinical program design should incorporate standard criteria for neoantigen vaccine trials, including exome and RNA sequencing for neoantigen identification, assessment of tumor mutational burden, HLA context, disease setting, safety profiling of checkpoint combination therapies, and evidence of antigen-specific T cell induction in Phase I studies [22,27]. It is advisable to establish antigen integrity throughout the manufacturing process and a specified burst-release specification prior to initiating IND-enabling preclinical work, rather than during it. Initial applications are reasonably contemplated in indications with an established infrastructure for immunotherapy combination therapies. Among the models utilized in the reviewed PLGA studies, triple-negative breast cancer (4T1) appears directly relevant [22,25], and colorectal cancer (CT26) is featured in the RGD-targeted lipid/PLGA study [23]. The extension to other immunotherapy-responsive indications, such as melanoma, is suggested based on standard-of-care considerations and route feasibility, rather than on direct efficacy data of PLGA nanoparticles in those indications.

### 7.2. MSNs: IND-Enabling Research Needed

bMSN formulations delivering CDN STING agonists represent a distinct development candidate for immunosuppressive tumor types akin to those examined in the reviewed studies, which support models encompassing melanoma and triple-negative breast cancer, rather than a broader disease spectrum [31,32,33]. Progression to clinical application necessitates IND-enabling repeat-dose toxicology studies in relevant species, with duration and species selection justified by formulation, route, schedule, and regulatory guidance; includes characterization of cytokine-release risk; validated CDN loading and release; biodegradation and excretion studies with mass balance for silicic acid; and GMP-compliant manufacturing processes [32,59].

### 7.3. MNPs: Device- and Formulation-Specific Characterization

MNP immunotherapy programs should prioritize formulation-specific safety measures beyond the ferumoxytol precedent, including validation of field dosimetry, characterization of heating heterogeneity, and a regulatory strategy for device–drug combinations. MRI-guided intratumoral delivery remains promising but technically demanding for anatomically accessible tumors. Establish registered combination trials with checkpoint inhibitors, building on the hyperthermia feasibility program [11] and the broader MNP immunotherapy literature [38]. Claims of superparamagnetism should be supported by magnetization measurements on the final formulation rather than assumed from composition [37].

### 7.4. COFs: Preclinical Research Priority

The COF formulations examined in the reviewed studies show broad mechanistic diversity, including photodynamic therapy, pyroptosis, ferroptosis, nanozyme catalysis, and TLS induction. This variety of mechanisms, especially in remodeling immunosuppressive tumor microenvironments, justifies ongoing preclinical investment; however, it does not indicate imminent clinical application. Immediate priorities encompass repeat-dose safety evaluations, biodistribution studies, the characterization of degradation products with mass balance conducted over extended periods of months rather than days, hemocompatibility assessments, immunogenicity testing, and the development of scalable, aqueous-phase, cGMP-compliant synthesis processes. Among the models employed in the reviewed COF studies, triple-negative breast cancer (4T1) is directly pertinent, and expansion to other immunosuppressed and combination-responsive contexts serves as illustrative guidance for the development of preclinical programs rather than as an evidence-based priority.

## 8. Limitations

The limitations of this review are stated explicitly and should be read as constraining every comparative statement made above.

The review constitutes a narrative critical synthesis complemented by a representative evidence map, rather than a systematic review. No protocol was registered before commencement; we reconstructed the search process by executing the documented PubMed strategy after drafting the narrative synthesis. Furthermore, the rule-assisted screening of titles and abstracts was not independently duplicated. Consequently, the flowchart of 568 records documents the reproducible audit search and the selection process for the representative evidence map without recreating every source encountered during the author’s preliminary review. The 233 reports deemed potentially relevant but not included in Table 2 were not classified as ineligible. The selection of the 15 primary reports was based on expert judgment, was not blinded, and prioritized mechanistic diversity, a specific comparator, a directly measured immune endpoint, and an in vivo antitumor effect. Selection bias remains possible, and the authors did not conduct a formal risk-of-bias assessment.

The study selection process was representative rather than comprehensive. Sixteen formulation-level records, derived from 15 primary in vivo reports, underpin the comparative framework. This represents a limited evidence base for comparing four material classes; therefore, the count-based thresholds outlined in the section Organization of the Evidence Map and Its Limitations are highly sensitive to the inclusion or exclusion of individual studies. The restriction to English-language publications excludes pertinent non-English literature. Furthermore, reliance on published reports introduces publication bias favoring positive results, which is likely significant in a field where negative formulation outcomes are seldom documented.

The evidence presented is predominantly preclinical and based on murine models. There are no direct comparative studies evaluating any two of the four platforms under identical conditions; consequently, all cross-platform assertions are derived from indirect comparisons across heterogeneous studies. Tumor Growth Inhibition (TGI) and related efficacy metrics are not directly comparable across sources due to differences in formulas, models, schedules, and comparators. Therefore, these metrics are reported descriptively and are not subject to pooling. The qualitative categories in Figure 3 serve as author-assigned descriptive indicators of the reviewed evidence base. Only one of the six dimensions includes a numerical decision rule; the remaining five rely on author judgment based on criteria that are not directly comparable, as outlined in the Section Organization of the Evidence Map and Its Limitations. These categories do not represent population-level estimates, are not aggregated into a composite score, nor do they rank efficacy. The four-level ordered presentation facilitates comparative reading; however, readers should interpret this with caution.

Several mechanistic claims in the source literature are inferred rather than confirmed by depletion or rescue experiments, as documented in Section 4.5, and this review inherits that uncertainty. Safety conclusions are constrained by the short observation windows typical of the source studies, particularly for COFs, where long-term biodegradation and repeat-dose data are absent rather than reassuring. Finally, the field is moving quickly, and the search closed on 20 June 2026, so formulations and trials reported after that date are not represented.

## 9. Future Perspectives

### 9.1. Artificial Intelligence-Assisted Nanoparticle Design

Formulation development within this domain largely remains empirical, with parameters such as particle size, surface charge, loading, and release optimized through iterative synthesis. Conversely, computational and machine-learning approaches offer alternative avenues, enabling prediction of particle size and charge based on composition and process parameters, estimation of encapsulation efficiency and release kinetics, modeling of interactions with biological membranes and fluids, and prospective formulation design to meet target-property specifications [60]. Three specific applications are directly pertinent to the platforms reviewed in this context. For PLGA, models that link process parameters to size distribution and encapsulation efficiency address the scale-up transfer challenge discussed in Section 2.1.2 by constraining the experimental scope required at the pilot scale. For MSNs, models correlating framework composition and degree of condensation with degradation kinetics could resolve the formulation-by-formulation degradation issue outlined in Section 2.2.1. For COFs, retrosynthetic and property-prediction models might identify aqueous-phase synthesis routes to frameworks with specified photophysical properties, which currently constitute the primary obstacle to cGMP compliance. In each instance, the foundational requirement is the availability of high-quality, consistent training data, which directly pertains to the reporting standards outlined in Section 6.3 [57]. Computational design capabilities, therefore, are inherently dependent on the quality of the literature on which they are based.

### 9.2. Personalized Nanomedicine

Personalization within this domain operates on two distinct levels that should not be conflated. Cargo personalization is more advanced: patient-specific neoantigens identified through exome and RNA sequencing can be encapsulated within a fixed carrier, a model in which PLGA-based vaccines are optimally suited, as the carrier remains a defined product while the payload varies [5,10]. Carrier personalization, in which particle properties are tailored to a patient’s tumor vasculature, stromal density, or myeloid composition, remains conceptual and faces a regulatory challenge that is seldom acknowledged. This challenge is that a carrier, which varies between patients, is difficult to characterize as a defined product. The pragmatic near-term approach involves utilizing a fixed, fully characterized carrier with a variable payload, complemented by patient selection rather than particle customization.

### 9.3. Biomarker-Directed Immunotherapy

The absence of predictive biomarkers constitutes a primary impediment to the rational selection of patients within nanoparticle immunotherapy programs. Three categories of biomarkers warrant development. Delivery biomarkers would identify patients whose tumors effectively accumulate nanoparticles, given the significant inter-patient and inter-lesion variability in accumulation, which remains largely unmeasured in most trials. Iron oxide nanoparticles are notably advantageous in this regard, as they offer an intrinsic imaging readout that could directly fulfill this purpose, an underexploited benefit of the magnetic nanoparticle (MNP) platform [36,55]. Target-engagement biomarkers would verify that the intended biological pathway is activated, such as the induction of type I interferon signatures for STING-directed formulations. This verification would distinguish pharmacological failure from inadequate delivery, as discussed in Section 2.2.2 [34]. Response biomarkers, including baseline T cell infiltration, PD-L1 expression, tumor mutational burden, and tertiary lymphoid structure (TLS) presence, would delineate the populations in which the conversion of a ‘cold’ tumor to a ‘hot’ tumor is feasible. Incorporating these measurements into early-phase trial designs is more likely to enhance success rates than further formulation optimization, since the attrition analysis in Section 6.4 primarily attributes failure to insufficient efficacy within unselected populations [8].

## 10. Conclusions

This critical comparative review synthesizes representative preclinical and translational evidence for four nanoparticle platform families used in cancer immunotherapy. The available evidence indicates distinct platform strengths and does not support a universal ranking. PLGA offers the strongest regulatory and manufacturing precedent at the polymer level, and its principal unresolved problems are antigen stability, burst release, and industrial process transfer. Biodegradable MSN formulations provide a strong rational-design basis for cytosolic STING agonist delivery, and their principal unresolved problems are formulation-specific degradation kinetics, long-term silica persistence, and immunotoxicity. MNPs combine imaging, field-assisted targeting, and hyperthermia, and their principal unresolved problems are the regulatory complexity of device–drug combinations, field dosimetry, and the non-transferability of the ferumoxytol precedent. Selected COF formulations show the broadest preclinical mechanistic range, but their principal unresolved issues are long-term biodegradation, degradation-product toxicology, and the lack of a cGMP-compliant synthesis route.

Advancing beyond platform-specific case studies toward clinical prioritization requires establishing standardized evidence. This entails consistent, transparent reporting of TGI data; incorporation of survival and immunological memory endpoints; execution of mechanistic depletion experiments; comprehensive biodistribution assessments with pharmacokinetic parameters; and repeat-dose toxicology studies. The evidence map presented in Table 1 and Table 2, along with Figure 3, delineates translational gaps and mechanistic distinctions across different platforms. It serves as a descriptive, hypothesis-generating tool and does not assign a composite score or assess clinical success probability. The mechanistic complexity observed in recent COF formulations reflects a broad spectrum of engineering capabilities rather than immediate clinical relevance and should be considered alongside the translational gap analysis detailed in Section 7.4.

The most significant translational gap is not a deficiency of promising mechanisms but rather the lack of standardized and comparable evidence. This issue is further exacerbated by an attrition pattern in which nanomedicines tend to fail at later stages, primarily due to insufficient efficacy rather than toxicity. Future research should consistently report tumor growth inhibition (TGI) using explicitly defined formulas, include endpoints such as survival and immune memory, assess immune-cell depletion whenever a mechanism is proposed, quantify biodistribution and clearance, perform repeat-dose toxicology studies, and establish the manufacturing process before IND-enabling preclinical investigations. Additionally, conducting comparative studies in immunocompetent, clinically relevant models is essential to develop a reproducible evidence base for clinical prioritization.

## Data Availability

No new experimental data were generated. Data were, however, extracted and qualitatively analyzed from the cited publications: the author selected studies against the eligibility criteria in Section 1.4, extracted formulation-level variables, assigned the descriptive categories shown in Figure 3, counted records per platform, and constructed Table 1 and Table 2 from those extractions. The formulation-level extraction table underlying Table 2 and Figure 3 is provided as Supplementary Table S1. No other datasets were created, and all primary data remain in the cited publications.

## Supplementary Materials

The following supporting information can be downloaded at: https://www.mdpi.com/article/10.3390/pharmaceutics18081019/s1, Supplementary Table S1 encompasses the formulation-level extraction records that support Table 2 and the descriptive evidence map illustrated in Figure 3. These include study identifiers and persistent source links.
